# Trend and heterogeneity in forced vital capacity among Chinese students during 1985–2019: results from Chinese National Survey on Students’ Constitution and Health

**DOI:** 10.1186/s12931-023-02573-5

**Published:** 2023-11-06

**Authors:** Siying Zhang, Lihong Wu, Yumei Zhong, Meirou Shao, Zhiyi Wei, Wenfeng Dong, Aiping Zhu, Fang-biao Tao, Xiulong Wu

**Affiliations:** 1 Department of Maternal, Child and Adolescent Health, School of Public Health, Anhui Medical University, No 81 Meishan Road, Hefei, 230032 Anhui China; 2 Key Laboratory of Population Health Across Life Cycle (Anhui Medical University), Ministry of Education of the People’s Republic of China, No 81 Meishan Road, Hefei, 230032 Anhui China; 3 Anhui Provincial Key Laboratory of Population Health and Aristogenics, Anhui Medical University, No 81 Meishan Road, Hefei, 230032 Anhui China

**Keywords:** Forced vital capacity, Chinese National Survey on Students’ Constitution and Health, Sex disparity, Urban–rural difference, Nationality heterogeneity

## Abstract

**Background:**

Forced vital capacity (FVC) reflects respiratory health, but the long-term trend and heterogeneity in FVC of Chinese students were understudied.

**Methods:**

Data were from Chinese National Survey on Students’ Constitution and Health 1985–2019. Super Imposition by Translation and Rotation model was used to draw FVC growth curves. Sex-, region-, and nationality-heterogeneity in FVC was evaluated. Spearman correlation and generalized additive model was used to reveal influencing factors for FVC.

**Results:**

Compared to 1985, age at peak FVC velocity was 1.09, 3.17, 0.74, and 1.87 years earlier for urban male, urban female, rural male, and rural female in 2019, respectively. Peak FVC velocity first decreased and then increased during 1985–2019, only male rebounded to larger than 1985 level. FVC declined from 1985 to 2005 and then raised. Males consistently had higher FVC than females, with disparities increasing in the 13–15 age group. Urban students also had higher FVC than rural students. In 2019, FVC difference between 30 Chinese provinces and the national average showed four scenarios: consistently above national average; less than national average until age 18, then above; greater than national average until age 18, then this advantage reversed; less than national average in almost all the age. Most Chinese ethnic minority students had lower FVC levels compared to Han students. Spearman correlation and generalized additive model showed that age, sex, and height were the leading influencing factors of FVC, followed by socioeconomic and environmental factors.

**Conclusions:**

Chinese students experienced advanced FVC spurt, and there was sex-, region- and nationality-heterogeneity in FVC. Routine measurement of FVC is necessary in less developed areas of China.

**Supplementary Information:**

The online version contains supplementary material available at 10.1186/s12931-023-02573-5.

## Introduction

Forced vital capacity (FVC) reflects airway function and respiratory health, and decreased FVC was associated with increased risks of respiratory, cardiovascular and renal disease [1–3]. Lung function follows a clear trajectory throughout the lifespan and can be divided into three distinct phases: a steep increase during childhood, a plateau at around 20–25 years old, followed by a gradual decline [4]. Thus, it is crucial to monitor lung function development from childhood to adulthood. Patterns of growth and peak level were the essential determinants of adulthood lung function, and prevention of reduced growth and/or early decline of FVC would help to improve overall health.

FVC is influenced by age, sex, height, ethnicity, and several environmental and socioeconomic factors, such as urbanization and air pollutants [5, 6]. In contrast to adulthood, FVC level during childhood and adolescence is characterized by monotonous increase rather than decline [7]. Additionally, due to sexual dimorphism in height and chest width during pubertal development, vital capacity develops disparately throughout the growth spurt [7]. Height is the main factor affecting lung function [5], and later puberty onset and faster height growth lead to higher FVC in young adults [8]. Ethnicity is a crucial factor that demands meticulous consideration in the evaluation of FVC. Latest guidelines from the American Thoracic Society and other professional organizations recommend utilizing ethnicity-specific reference equations to interpret lung function test results [9]. Chinese were reported to have lower FVC than North Americans and Europeans [10]. Yan et al. conducted a study of ethnic differences in lung function in Chinese aged 35–70 years and showed that among four ethnic groups studied, only Mongolian had higher FVC than Han, while Uygur, Hui, and Dai had similar or lower FVC than Han [11]. However, there is little research on Chinese minority children and adolescents. Rapid urbanization and socioeconomic level have also been identified as important factors for cardiorespiratory health, but research findings were inconsistent. For example, results from cohort studies in the United Kingdom showed a positive correlation between socioeconomic level and FVC [6], but findings of the study performed in Lanzhou, China were opposite [12]. Recent global studies revealed a decline in cardiorespiratory fitness among children and adolescents. Data from 14 countries (including China, Finland, and Sweden) demonstrated a continuous decrease in lung capacity and fitness levels during 1969–2017 [13]. Dong et al. explored trends in physical fitness and the effects of nutritional transition on physical fitness in Chinese students during 1985–2014 [14], although FVC was an important component of physical fitness, they did not comprehensively reveal the change in FVC growth patterns or potential influencing factors. Given China’s rapid urbanization and vast regional disparities, it is essential to explore the latest characteristics of FVC among Chinese students.

We aimed to analyze changes in FVC growth pattern, and explore sex and urban-rural differences of FVC among Chinese students during 1985–2019. Besides, FVC gaps between 30 provinces and the national average, between 26 ethnic minorities and Han nationality students, as well as potential influencing factors of FVC levels were further studied.

## Methods

### Study participants

In this analysis, we obtained data from eight successive Chinese National Survey on Students’ Constitution and Health (CNSSCH) conducted in 1985, 1991, 1995, 2000, 2005, 2010, 2014, and 2019. The CNSSCH is the largest comprehensive survey of the health status of Chinese students aged 7–22 years old, covering 30 provinces, municipalities, and autonomous regions (excluding Hong Kong, Macau, Taiwan, and Tibet of China). Randomly selected schools from different provinces were surveyed, and the sampling was stratified by grade, with random whole-group sampling by teaching class. Notably, there were no data for Hainan and Chongqing in 1985 and 1991, and no data for Qinghai and Chongqing in 1995. The surveys included both urban and rural (urban students were those of agricultural Hukou, and rural students were those of non-agricultural Hukou; college students were classified by Hukou type before college entrance), and male and female students, as well as Han and minority. For CNSSCH 1985–2019, 2,253,530 Chinese Han students aged 7–22 years old were analyzed. In 2019, 256,032 Chinese Han students and 89,752 minority students aged 7–18 years were used for cross-sectional analysis. In the survey of Chinese minority students, schools were randomly selected from ethnic minority communities in different provinces. Region-, sex-, and age-specific sample size for Chinese Han students was presented in Table 1 and Additional file 1: Table S1 and sex- and age-specific sample size for each minority group was shown in Additional file 1: Table S2. Students and their guardians consented to participate in the survey, and the research protocol was reviewed and approved by the Medical Research Ethics Committee of the Peking University Health Science Center (No. IRB00001052-19095).

**Table 1:** Characteristics of Chinese students aged 7–22 years old in the eight CNSSCH, from 1985 to 2019

Age (years)	1985	1991	1995	2000	2005	2010	2014	2019
Urban	245,688	93,545	128,150	133,497	147,118	131,272	130,837	128,542
7	17,119	5858	8800	8925	9664	8967	8934	8962
8	17,122	5976	8803	9064	9699	8953	8969	9013
9	17,118	5910	8799	9063	9780	8970	8966	9057
10	17,116	5874	8792	9236	9807	8976	8971	8896
11	17,120	5900	8809	9021	9912	8995	8959	9046
12	17,115	5832	8794	9041	9682	8967	8948	9049
13	17,116	5839	8781	9076	9826	8972	8979	8942
14	17,121	5879	8782	9058	9708	8982	8970	9032
15	17,112	5876	8794	9084	9913	8971	8967	8922
16	17,116	5847	8737	9069	9806	8929	8971	8774
17	17,069	5846	8797	9022	9772	8971	8983	8808
18	16,483	5901	8699	9179	10,022	8909	8576	8167
19	11,067	5925	5711	6755	7712	5966	5970	5607
20	11,173	5921	5749	6486	7625	5956	5957	5736
21	11,054	5521	5745	6146	7474	5957	5974	5507
22	7667	5640	5558	5272	6716	5831	5743	5024
Rural	231,427	91,220	125,934	132,718	143,574	131,549	130,969	127,490
7	17,084	5771	8693	9103	9360	8965	8935	9062
8	17,118	5861	8698	9017	9427	8969	8881	8905
9	17,117	5876	8678	8969	9523	8980	8973	8878
10	17,121	5760	8711	9043	9763	8981	8968	9099
11	17,120	5855	8700	8967	9617	8981	8955	8952
12	17,123	5832	8688	9010	9543	8994	8946	8879
13	17,114	5838	8645	8863	9580	8973	8967	8783
14	17,115	5844	8681	8943	9575	8977	8970	8708
15	17,117	5888	8679	8934	9834	8985	8988	8908
16	17,114	5893	8606	8957	9740	8971	8958	8780
17	17,116	5878	8604	8859	9732	8952	8974	8698
18	16,960	5821	8546	9102	10,280	8977	8548	8312
19	11,607	5273	5375	6421	7353	5973	5976	5433
20	5569	5676	5575	6345	7173	5992	5977	5642
21	5223	5226	5583	6533	6769	5,986	5984	5601
22	3809	4928	5472	5652	6305	5893	5969	4850

Data were number of people; CNSSCH, Chinese National Survey on Students’ Constitution and Health

### FVC measurement

Following *Rules of Chinese students’ Constitution and Health Survey*, FVC testing methods remained consistent across eight successive surveys, but three types of spirometers were used in different surveys (Additional file 1: Tables S3, S4). Technicians were trained and they adhered to a standardized protocol to ensure accuracy. Spirometer met specific technical criteria, ensuring an acceptable margin of error ≤ 3%. Furthermore, environmental factors, such as room temperature, atmospheric pressure, and humidity, were appropriately adjusted for body temperature, pressure and saturated with water vapor.

### Evaluation of influencing factors

We retrieved the age, sex, and height of Han students from CNSSCH 2019. Longitude and latitude information of each province (municipality) came from China National Geographic Information Center. Park green space areas and urbanization rate of 2019 were retrieved from the Chinese Statistical Yearbook 2020. Gross Domestic Product (GDP) per capita of 2019 was retrieved from the website of the National Bureau of Statistics of China (https://data.stats.gov.cn/index.htm). The annual China regional-level mean particulate matter with aerodynamics diameter < 2.5 μm (PM_2.5_) data came from the Atmospheric Composition Analysis Group, Washington University in St. Louis, USA (https://sites.wustl.edu/acag/datasets/surface-pm2-5/) and we used PM_2.5_ data in 2019.

### Statistical analyses

FVC growth curves were plotted using the Super Imposition by Translation and Rotation (SITAR) model, a shape-invariant growth model that provides a summary growth curve and undergoes three transformations. In comparison to other fitting methods, such as Preece–Baines growth curves, this approach presents a more efficient alternative by employing the fitting of a single curve for each individual. A natural cubic regression *β*-spline with fixed-effects and specified degrees of freedom, 3 was selected as optimal degree of freedom according to the Bayesian information criterion and Akaike information criterion in our study [15]. Three transformations were used to convert complex FVC data into three clinically relevant parameters that represented the size, tempo, and velocity of growth, which was not available in the random intercept—random slope model, and it is the innovation of SITAR. Three transformations were used to convert complex FVC data into three clinically relevant parameters that represented the size, tempo, and velocity of growth. Size parameter reflected the upward and downward shift of the mean curve, indicating whether an individual’s FVC was greater or less than the mean. Tempo parameter indicated the left and right shift of the mean curve, indicating whether an individual’s age of FVC peak velocity was earlier or later than the mean. Velocity parameter reflected the stretching and shortening of the average curve, indicating whether an individual’s FVC growth curve was flatter or steeper than the average [15]. Age at peak FVC velocity (APFV) was estimated using the tempo parameter, and peak FVC velocity (PFV) was assessed using the velocity parameter. APFV and PFV in 1991, 1995, 2000, 2005, 2010, 2014, and 2019 were compared with the values in 1985 using independent samples *t* test. Chinese Han students were divided by age (7–9, 10–12, 13–15, 16–18, 19–22 years old) [14], sex (male and female), and region (urban and rural) to compare the trend in FVC among different subgroups. Absolute increase of FVC was the value of the observed year minuend the value of the initial year (1985) and the transition year (2005). Increase rate of FVC was the ratio of absolute increase to the initial year (1985) and the transition year (2005). Additionally, comparisons were made between Chinese Han students and national average by province in 2019 (in Qinghai, data of 19- and 22-year-old urban male, and 19-, 21-, and 22-year-old rural male were missing), as well as between Chinese minority and Han students. To assess the relationship between potential influencing factors (age, region, height, GDP per capita, urbanization rate, longitude, latitude, PM_2.5_, and park green space) and FVC, Spearman correlation and generalized additive model (GAM) were performed. Age, height, GDP per capita, urbanization rate, longitude, latitude, PM_2.5_, and park green space were included as smooth terms and independent variables with adjustments for region and sex.

Analyses of growth curves were conducted using R “SITAR” and “nlme” packages, and GAM using R “mgcv” package (version 4.2.2, R core team, Vienna, Austria). Two-sided *p* < 0.05 was regarded as statistically significant.

## Results

### Trends in APFV and PHV of Chinese Han students from 1985 to 2019

**Fig. 1:**
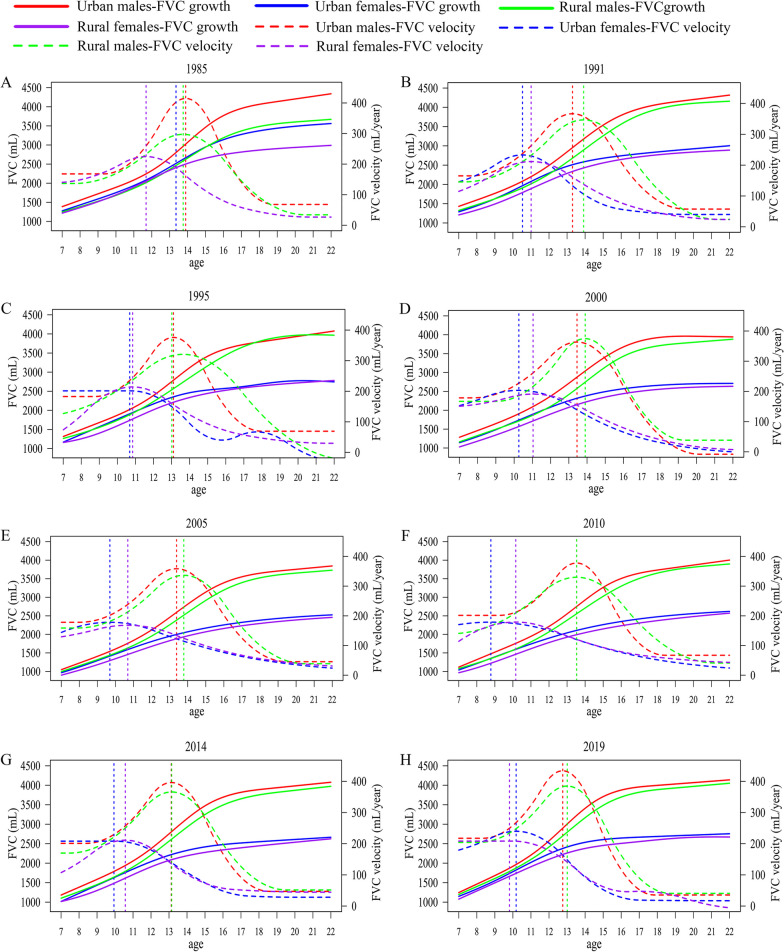
Sex and urban-rural differences in the FVC spurt curve of Chinese Han student aged 7–22 years by SITAR model, in 1985 (**A**), 1991 (**B**), 1995 (**C**), 2000 (**D**), 2005 (**E**), 2010 (**F**), 2014 (**G**) and 2019 (**H**). *FVC* forced vital capacity, *SITAR* Super Imposition by Translation and Rotation. All lines in the figure were generated by the SITAR model. The solid curves represented FVC growth curves; the dashed curves represented FVC velocity curves; the dashed vertical lines represented the location of the surge age

For eight successive CNSSCH, numbers of Han students included in each survey were 477,115, 184,765, 254,084, 266,215, 290,692, 262,821, 261,806, and 256,032, respectively (Table 1 and Additional file 1: Table S1). Figure 1 displayed the FVC spurt curve of Chinese Han students aged 7–22 from 1985 to 2019, stratified by sex and region. FVC was generally higher in male than that in female, and higher in urban students than rural. Both male and female experienced an FVC spurt, with sex difference in FVC level enlarged after APFV. Notably, female had earlier APFV than male, regardless of whether they lived in urban or rural area. However, in 1985 and 2019, female in rural area showed earlier APFV than their urban counterparts. During 1985–2019, APFV declined from 13.85 to 12.76 years old in urban male, 13.35 to 10.18 in urban female, 13.75 to 13.01 in rural male, and 11.68 to 9.81 in rural female (Fig. 1 and Additional file 1: Table S5, all *p* < 0.001). In 2019, rural male had the latest APFV among four groups, followed by urban male. Additionally, PFV was significantly higher in male than female in all eight surveys (*p* < 0.001). Urban student had higher PFV than rural, and PFV of urban male, urban female, and rural female all showed an upward trend after 2005, while rural male’s PFV rebounded after 2010. Besides, male student had higher PFV in 2019 than 1985 (urban: 433.98 vs. 413.40 mL/year; rural: 384.97 vs. 297.40 mL/year; Additional file 1: Table S5), but PFV of female students did not rebound to 1985 level.

### Trend in FVC of Chinese Han students by age, sex, and region from 1985 to 2019

FVC in five age groups of urban male (*n* = 570,748), urban female (*n* = 567,902), rural male (*n* = 557,930), and rural female (*n* = 556,951) showed a “V-shaped” trend, declined from 1985 to 2005 and increased thereafter (Additional file 1: Figure S1 and Table S6). Urban boys aged 10–12 and 13–15 years demonstrated an increased rate of 7.79% and 10.21% in FVC, respectively, compared 2019 with 1985, while all other age groups exhibited a decreasing trend, and the 7–9 age group showed the most substantial decline rate. Similar results were observed for rural boys (Additional file 1: Table S6, Figure S1A and C). In 2019, only girls aged 10–12 showed an increase in FVC compared to 1985, and the largest decline rate was observed in females aged 19–22 years (Additional file 1: Table S6, Figure S1B and D).

### Heterogeneity in FVC of Chinese Han students by age, sex, and region from 1985 to 2019

**Fig. 2:**
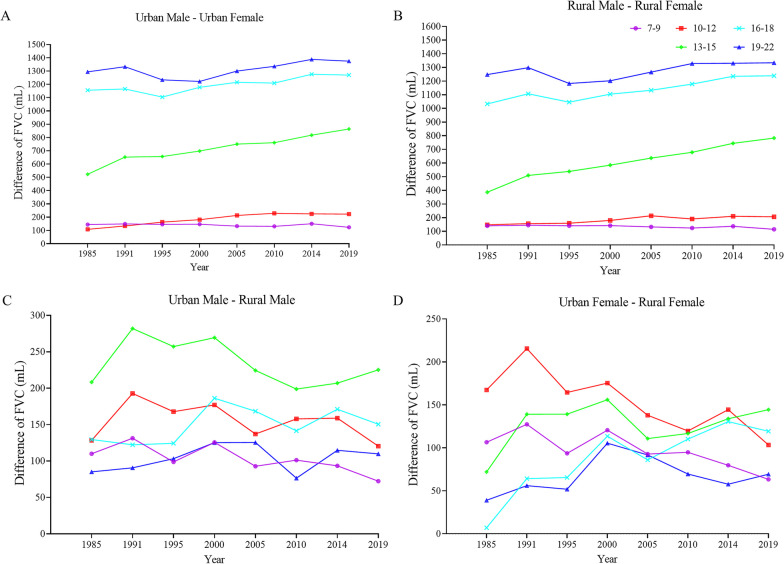
Trends in difference of FVC in Chinese Han students from 1985 to 2019, by sex, region, and age groups. *FVC* forced vital capacity. **A** Trends in difference of FVC between urban male and female by age group, 1985 to 2019; **B** trends in difference of FVC between rural male and female by age group, 1985 to 2019; **C** trends in difference of FVC between urban male and rural male by age group, 1985 to 2019; **D** trends in difference of FVC between urban female and rural female by age group, 1985 to 2019

FVC heterogeneity between male and female increased with age, regardless of the Hukou type. FVC difference between male and female in 19–22 years was almost 9–11 times higher than that of children aged 7–9 years (Fig. 2A and B, and Additional file 1: Table S7). Additionally, there was a sharply upward trend in sex discrepancy of FVC in age group 13–15 years from 1985 to 2019 (Fig. 2A and B). FVC of urban students consistently exceeded rural students throughout eight surveys, and the largest urban-rural difference in FVC was observed in male aged 13–15 years and female aged 10–12 years. Nevertheless, it was worth noting that in 2019, urban–rural difference for female in the 13–15 age group exceeded that of the 10–12 age group (Fig. 2C and D).

### Difference in FVC between Chinese Han students from 30 provinces and national average in 2019

**Fig. 3:**
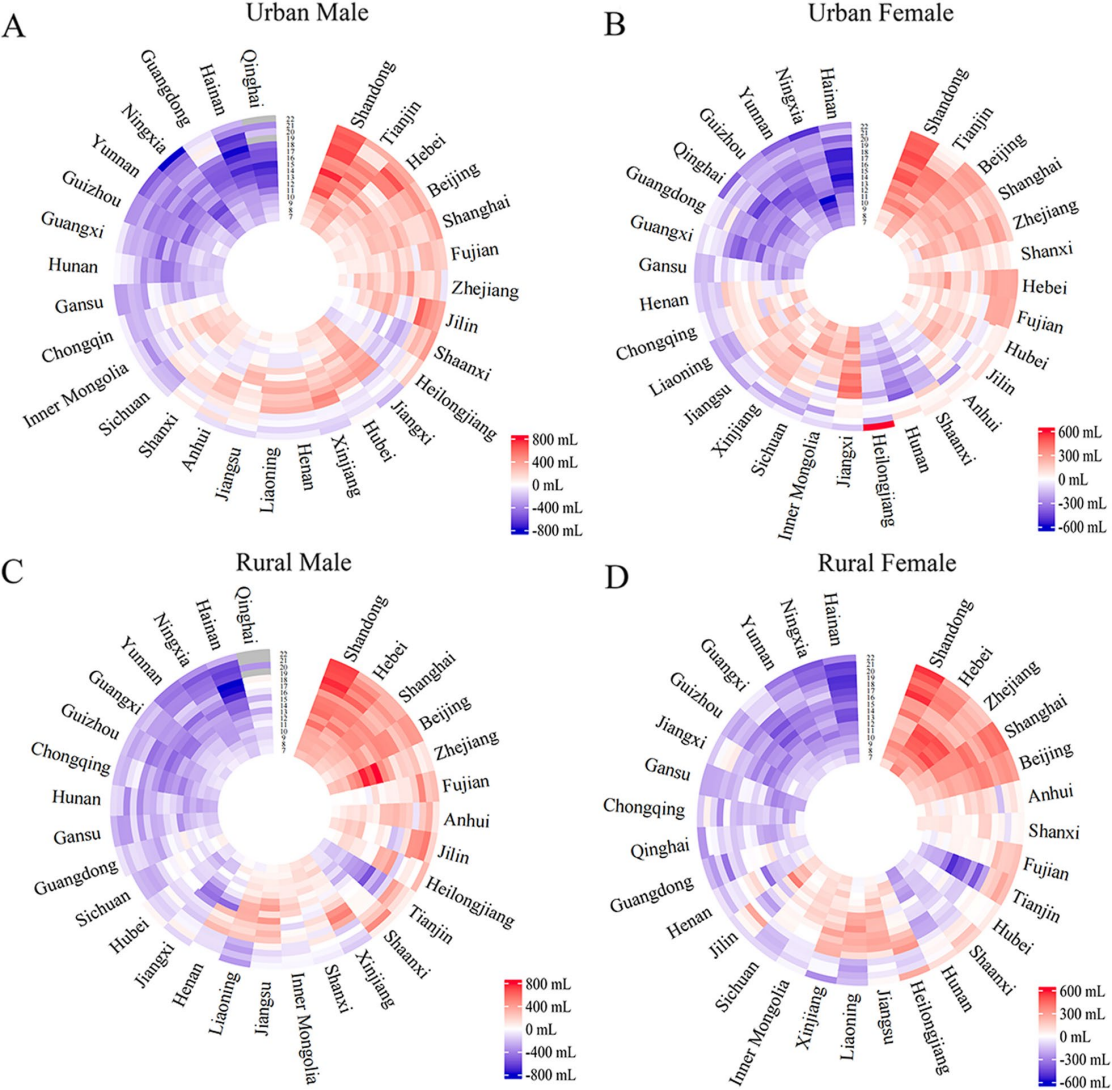
Heterogeneity in FVC across 30 Chinese provinces in Chinese Han students in 2019, by sex and region. *FVC* forced vital capacity. **A** Difference between FVC of urban male Han students in each province of China and the national average in 2019 (data of Qinghai missing for 19- and 22-year-old); **B** difference between FVC of urban female Han students in each province of China and the national average in 2019; **C** difference between FVC of rural male Han students in each province of China and the national average in 2019 (data of Qinghai missing for 19-, 21-, and 22-year-old); **D** difference between FVC of rural female Han students in each province of China and the national average in 2019. The words in the outer circle represented the 30 provinces surveyed in 2019 in the mainland of China

We compared the FVC of Chinese Han students aged 7–22 years in 30 provinces with age-specific national average in 2019. We identified four distinct scenarios that captured FVC patterns among different provinces, using an 18-year demarcation point. (1) Greater than the national average in almost all the age, like Beijing, Shandong, Shanghai, and Zhejiang. Notably, the advantages in FVC of the students in Shandong Province expanded with age increase in four subgroups. (2) Less than the national average until age 18, then this disadvantage reversed: urban male in Shaanxi and Heilongjiang, urban female in Anhui, Shaanxi, Hunan, and Heilongjiang, rural male in Shaanxi and Tianjin, and rural female in Tianjin, Shaanxi, Hunan, Hubei, and Heilongjiang. (3) Greater than the national average until age 18, then this advantage reversed: e.g., Liaoning, Jiangsu, Sichuan, and Xinjiang. (4) Less than the national average in almost all the age: students in the Gansu, Guizhou, Yunnan, and Hainan (Fig. 3, Additional file 1: Figure S2 and Table S8).

### Difference in FVC between Chinese minority and Han students in 2019

**Fig. 4:**
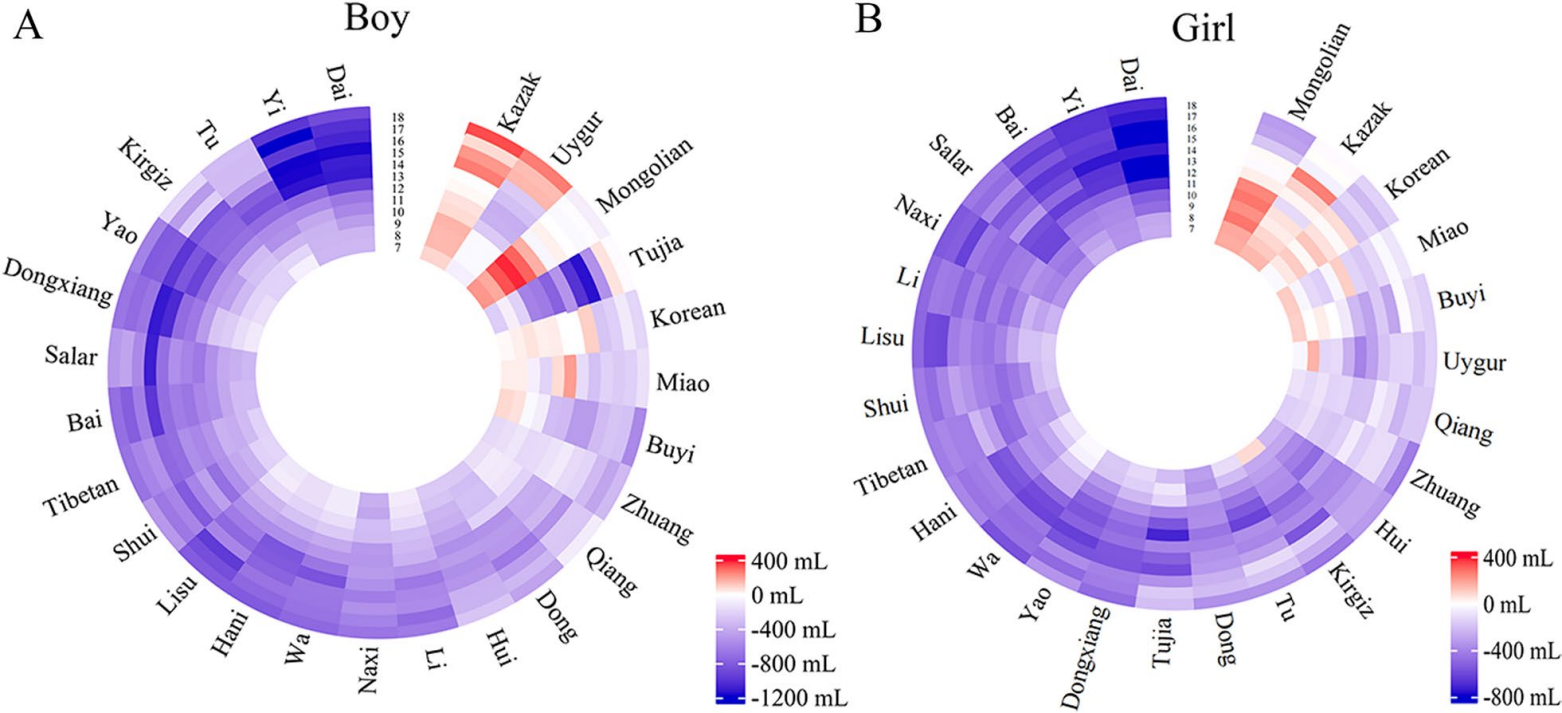
Heterogeneity in FVC between Chinese ethnic minorities and Han nationality in 2019, by sex. *FVC* forced vital capacity. **A** Difference in FVC of Chinese boys between ethnic minorities and the mean of Han nationality in 2019; **B** difference in FVC of Chinese girls between ethnic minorities and the mean of Han nationality in 2019. The words on the outer ring represented the 26 ethnic minorities surveyed in China in 2019

In 2019, compared FVC levels of Chinese minority students with Han, results showed that most ethnic minorities had smaller FVC levels (Fig. 4 and Additional file 1: Table S9). Among the 27 nationalities surveyed in 2019, the Han nationality consistently ranked higher in terms of FVC levels across all age groups (Additional file 1: Figure S3). Kazak boy showed a slight FVC deficit compared to Han boy at 14 years, but displayed an FVC advantage at all other ages. Similarly, Uygur boy exhibited an FVC disadvantage before 16 years of age, then reversed. In contrast, Mongolian, Korean, and Miao boy had higher mean FVC levels than Han boy throughout childhood and early adolescence, but a deficit was observed in late adolescence (Fig. 4A and Additional file 1: Table S9). The heterogeneity between ethnic minority girl and Han girl was even more striking, with no ethnic minorities displaying steadily higher FVC levels than Han girl across all age groups. Mongolian, Kazak, and Korean girl showed FVC advantages from ages 7 to 15 years, but this advantage disappeared thereafter (Fig. 4B and Additional file 1: Table S9). Dai and Yi students were observed to have the lowest FVC levels across most age groups, particularly in the 13–15 years (Additional file 1: Figure S3 and Table S9).

### Potential influencing factors of FVC among Chinese Han students in 2019

**Fig. 5:**
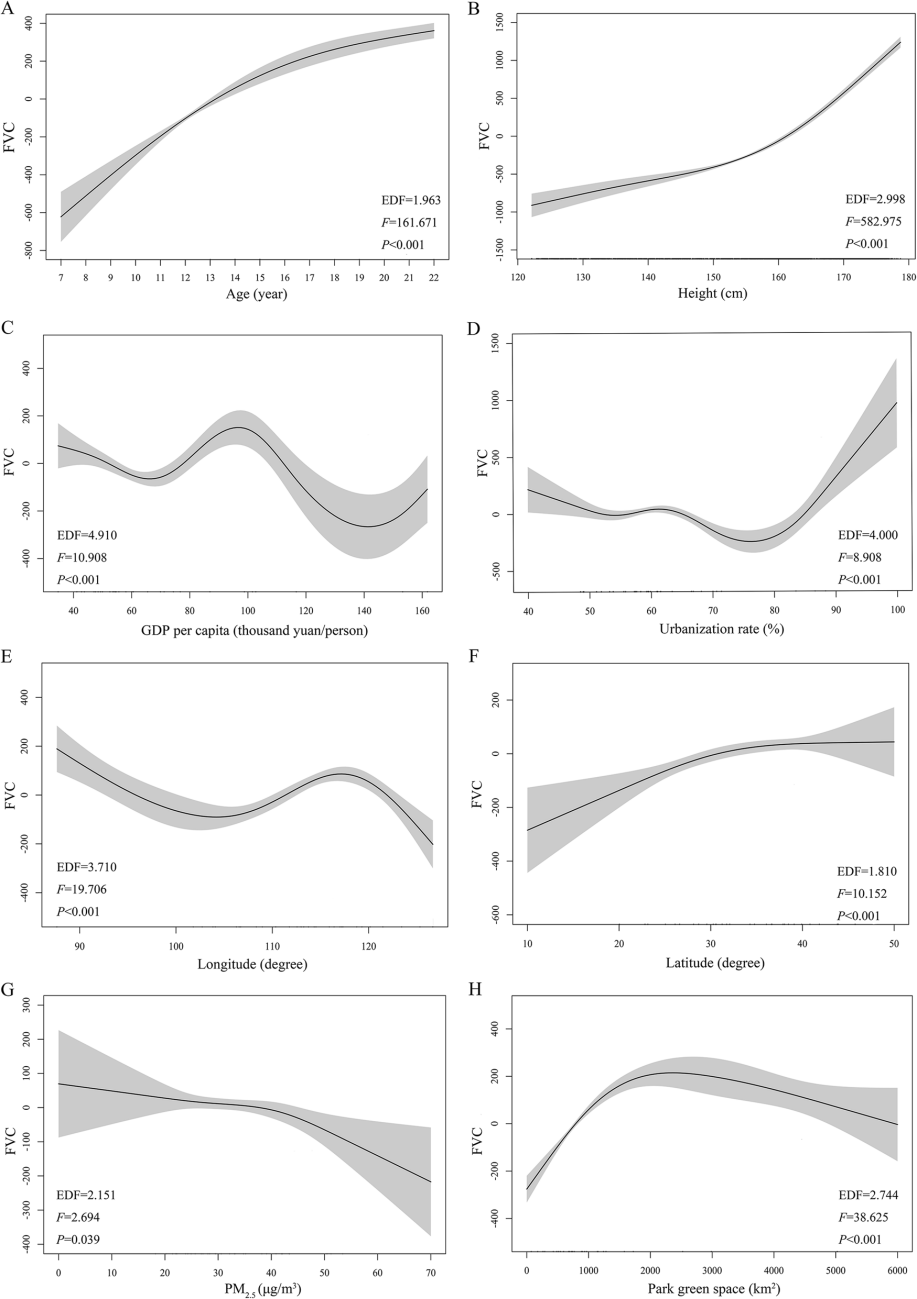
Relationships of influencing factors with Chinese Han students’ FVC in 2019 using GAM. *FVC* forced vital capacity, *GAM* generalized additive model, *PM*_2.5_ particulate matter with aerodynamics diameter < 2.5 μm. **A** Effect of age on FVC in the multivariable GAM; **B** Effect of height on FVC in the multivariable GAM; **C** Effect of GDP per capita on FVC in the multivariable GAM; **D** Effect of urbanization rate on FVC in the multivariable GAM; **E** Effect of longitude on FVC in the multivariable GAM; **F** Effect of latitude on FVC in the multivariable GAM; **G** Effect of PM_2.5_ on FVC in the multivariable GAM; **H** Effect of park green space on FVC in the multivariable GAM. The values of PM_2.5_ in the table represented the annual mean PM_2.5_ value in 2019; GAM model was adjusted for sex and area; adjusted R-Squared for this model was 0.937 and deviance explained was 93.7%

To further explore the reasons for the provincial differences, we conducted Spearman correlation and GAM analysis about FVC of students in 2019 (Additional file 1: Table S10 and Fig. 5). FVC level was strongly correlated with height (*r* = 0.943, *p* < 0.001), followed by age and sex (*r* = 0.802 and −0.363, respectively, both *p* < 0.001; Additional file 1: Table S10). FVC level was weakly correlated with area (*r* = −0.077), GDP per capita (*r* = 0.103), urbanization rate (*r* = 0.111), longitude (*r* = 0.142), latitude (*r* = 0.094), PM_2.5_ (*r* = 0.126) and park green space (*r* = 0.143, Additional file 1: Table S10). We further performed GAM and found nonlinear relationships between the influencing factors and FVC (adjusted *R*^2^ = 0.937 for the GAM model, and effective degree of freedom of each factor was not around 1). There were positive associations of age and height with FVC. GDP per capita and FVC had a “W-shaped” relationship, and FVC initially decreasing and then increasing with urbanization rate increasing. We observed that FVC showed a pattern of initially decreasing, then increasing, and again decreasing with longitude, and a consistent increasing trend with latitude. Additionally, there was a decreasing trend in FVC associated with PM_2.5_, and the decline accelerated after > 40 µg/m^3^. However, after park green space reaching 2000 km^2^, improvement trend of FVC stagnated or even exhibited a decline with further raise (Fig. 5). When fitting GAM by sex, relationships of influencing factors with FVC in the male students were similar to those in the whole population. But for female students, there was an inverse “N-shaped” relationship between GDP per capita and FVC, and FVC initially increased and then slightly decreased with urbanization rate increasing. Besides, there was a nearly “N-shaped” relationship between park green space area and FVC among females (Additional file 1: Figures S4, S5).

## Discussion

Our study utilized the latest national and provincial data to explore long-term trends and heterogeneity in FVC among Chinese students aged 7–22 years from 1985 to 2019. We found that in Chinese students, APFV in 2019 was earlier than that in 1985, with female experiencing further advancement than male. During the 35 years, FVC exhibited a “V-shaped” trend, sex differences in FVC increased, and urban students exhibited greater FVC than rural students. In 2019, the age trajectory of FVC growth in 30 provinces could be divided into four scenarios, and most Chinese ethnic minority students had smaller FVC than Han. FVC level was dominantly influenced by age and height, followed by socioeconomic and environmental factors. A comprehensive understanding of growth pattern and influencing factors of FVC is essential to formulate effective strategies to improve respiratory health of Chinese students.

APFV has not been extensively studied in children. We found that compared to 1985, APFV was earlier in 2019, especially for female. This shift may be due to an earlier onset of puberty, which has been a long-term trend and along with earlier height spurt. Chinese Han girls have shown an advanced trend in age at menarche, decreasing from 13.03-year-old in 1995 to 12.00-year-old in 2019 [16]. Spermarche in Han boy has also begun earlier (14.6-year-old in 1995 vs. 13.9-year-old in 2019), especially for rural boy, which may be related to enhanced prevalence of childhood obesity [17]. Because adequate store of fat and energy would allow earlier onset of puberty [18]. We also found that female had larger decrease in APFV than male, which would be a result of sex difference in the advancement of puberty onset. Male has higher height than female in almost all the time of childhood, later puberty onset and higher velocity of height growth led to greater attainment of adulthood lung function in male [8, 19]. Our results also showed that male had later APFV, larger PFV, and higher FVC than female. More studies are needed to elucidate secular trends of FVC in children and adolescents.

We observed that age-specific FVC declined from 1985 to 2005, improved during 2005–2019 for both sexes and regions, but most age groups did not rebound to 1985 level. This finding was similar to previous results [14], and FVC levels in children and adolescents in Xinjiang, China, were also observed to reach a minimum in 2005 [20]. We updated the data to 2019, included eight successive CNSSCH, and expanded it to be nationwide. FVC decline of Chinese Han children may be related to insufficient physical activity, obesity and air pollution [21, 22]. In 2005, a survey in China revealed that over 60% of students aged 13–18 did not have enough time for physical activities [20]. According to a 2016 survey, only 29.9% of students followed the recommended daily physical activity standards [23]. To address this issue, the Chinese government introduced policies like the National Program for Child Development (2001–2010) and the Suggestions on Strengthening Adolescents’ Sports (2007). Besides, the air pollution in China has undergone significant amelioration since 2005, as evidenced by an appreciable 8.1% reduction in sulfur dioxide emission and a concomitant 1.6% decline in PM_2.5_ emissions from 2006 to 2010 [24]. China has been working towards improving urban landscape, which could create more green spaces and enhance physical activity, helping to improve lung function [25, 26]. All these may help to explain the rebound in FVC among Chinese Han students after 2005. Interestingly, male aged 13–15 years old and female aged 10–12 years old showed largest FVC increment during 1985–2019, possibly due to difference in age-specific increment in height [27]. Secular trend in FVC resulted from the complex interaction between physical activity, nutrition, air pollution, and greenness.

Our study found that sex difference in FVC expanded in 13–15 years old, and urban students exhibited greater FVC than rural students. Growth rate of trunk during prepubertal and pubertal periods was different by sex. Although age of puberty onset decreased in both boys and girls, but more obvious in females than males [16, 17], so the development potential of FVC in female was less than male, and the gap increased year by year. For Chinese urban children and adolescents, the largest sex difference in height increment was also observed at around 14-year-old during 1975–2005 [27]. Persistent height advantage of urban students over the past 30 years is part of the explanation of urban-rural heterogeneity in FVC [28]. Studies of urban-rural differences in physical fitness of children and adolescents have been conducted in many countries. For instance, cardiopulmonary fitness among Mexican urban children and adolescents was found to surpass that of their rural counterparts [29]. Conversely, physical fitness levels were lower among children and adolescents in urban areas of Austria compared to those living in rural regions [30]. Interestingly, no disparity in cardiopulmonary fitness was observed between rural and urban adolescents in Kosovo [31]. These inconsistent findings may be attributed to differences in culture and urban-rural economic gap among these countries. Construction of livable and healthy cities and villages requires appropriate urban planning and infrastructure to promote physical activity. This is especially important in the prevention and control of chronic respiratory diseases.

​FVC difference between Han Chinese students and the national average showed four patterns, and most Chinese ethnic minority students had smaller FVC than Han. In 2019, the spatial distribution of FVC across 30 Chinese provinces presented a trend of “high in the North and low in the South, high in the East and low in the West”. A study revealed that students in the Chinese eastern region generally had higher heights than their western peers during six CNSSCH surveys (1985–2010), both in rural and urban settings [32]. In addition, numerous studies have provided evidence that air pollutants the adversely affected lung function of children, both in short-term and long-term exposures [22]. An examination of air pollutants in 336 Chinese cities from 2014 to 2019 revealed that air quality index was highest in northwestern and northern China, as well as in Henan province [33]. Several studies have shown that environmental pollutants have a lag effect on lung function, which continues to affect childhood growth and development [34]. This may explain the widening age-related gap in FVC between northwest China and the national average. What’s more, Northwestern China is also located at a higher altitude, and some studies have shown a negative correlation between vital capacity and altitude in people aged 6–18 years, as the lung compensatory mechanism was not fully developed [35]. We found that vast majority of Chinese minority students had lower FVC than Han students in 2019. A study by Pan et al. found that boys from North Korea and Kazakhstan had higher FVC levels than the national average, while girls from Korea, Tujia, Kazakh and Yi ethnic groups also exceeded the national average [36]. In contrast, we found only Kazak students consistently surpassed the Han average in all age groups, while other ethnic groups exceeded the Han average only in some specific age groups. The 26 ethnic groups covered by CNSSCH 2019 were mainly located in the Yunnan-Guizhou Plateau, Sichuan Basin, and Wuling Mountains, which are underdeveloped regions of China. Lung function varies significantly among ethnic groups due to body size, socioeconomic status, and environmental factors. Sitting height and chest width accounted for 16% of ethnic variation of lung function, and growth rate and timing of pubertal maturation also varied among ethnic groups [37]. Yan et al. discovered that among five nationalities in China aged 35 to 70, only Mongolians exhibited greater FVC than Han, and Dai had the lowest FVC [11]. Although the age of the study population differed, our findings also suggested that Mongolian boys and girls aged 7–15 years had larger FVC than Chinese Han counterparts, but Dai and Yi had relatively lower FVC throughout childhood. ​Ethnic differences in lung function arise from a complex interplay of genetic, socioeconomic and environmental factors. To address the developmental disparities in China, China implemented strategies like the “West China Development” policy. Due to the large population, small shifts in disease prevalence have a large impact on the number of patients. Developing ethnicity-specific reference equations is critical for accurate lung function prediction and reducing misdiagnosis of respiratory diseases.

We confirmed the strong association between height and FVC, genetic factors determine growth potential. Relationship between socioeconomic status and students’ FVC was complex. Findings of meta-analysis of 33 papers showed that children with disadvantage socioeconomic circumstances had lower lung function, and respiratory health inequalities were higher in boys than girls [38]. Another study included seven successive Chinese National Survey on Students’ Constitution and Health, and they found the “U-shaped” relationships of GDP per capita and urbanization rate with FVC in both boys and girls by using quadratic analysis [39]. In our study, GAM was performed, and ranges of GDP per capita and urbanization rate were larger than previous study [39], which would enable us to capture more complex patterns of FVC change with improvement of socioeconomic circumstance, like “W-shaped” and inverse “N-shaped” curves, not merely “U-shaped” curve. Sex-specific relationship of urbanization rate with FVC were also identified in our study, further study conducted in the Asian population would help to confirm our findings. Latitude and longitude determine geographical location, which affects students’ socioeconomic status and exposure to different environmental factors. Research has confirmed that the detrimental effects of air pollution on lung function and respiratory health [22]. These effects were attributed to various mechanisms, such as the induction of airway inflammation and oxidative stress [40]. Therefore, it is crucial to reduce air pollution levels and implement measures to improve air quality in order to safeguard lung function. The impact of greenness on children’s lung function remains debated. Evidence from the Avon Longitudinal Study of Parents and Children birth cohort suggested that residential greenspace has a positive effect on lung function levels at 8, 15, and 24 years old [41], but a study of 3,428 Norwegian and Swedish participants showed that increased normalized difference vegetation index was associated with lower FVC [42]. Boeyen et al. observed no significant association between surrounding greenness and lung function among 360 school children from Western Australia [43]. We found an inverse “V-shaped” relationship of park green space area with FVC in the whole population and males, but nearly “N-shaped” relationship in the females. Different measurement methods of greenness may produce different results. Increasing in park green space led to improvement in air quality, and increased levels of biodiversity and physical activity [44]. On the other hand, allergic risk increased with green space area increase due to pollen plants. As known, females have smaller lung size, narrower airway diameter, and shorter expiratory time constant than males. Sex difference in the relationship of park green space with FVC may result from sex difference in physiological characteristics of lung. Interaction of these factors has contributed to the inter-provincial FVC variation in China. Thus, implementation of regional policies aimed at reducing unfavorable environmental and economic disparities is a necessary condition for improving lung function.

Present study had a number of notable strengths. Firstly, it stood out as the first study to examine trend and heterogeneity in FVC among Chinese students from 1985 to 2019, a 35-year period marked by rapid urbanization and industrialization in China. Notably, nationally representative survey with large sample size improved the credibility of our findings. Secondly, we applied SITAR model to investigate the age and velocity of FVC spurt, making it the first to undertake such an investigation among children and adolescents. Lastly, this study considered not only the latest age-specific FVC level of Chinese Han students but also 26 minorities, thereby enriching known evidence. Our study also had following limitations. Firstly, CNSSCH was a cross-sectional study rather than a large, high-density cohort study. To some extent, our findings reflect long-term trends that cannot be accurately measured [17], but it can help to study FVC growth and development in Chinese children and adolescents. Secondly, types of spirometers used for the eight surveys of FVC were not the same, but strict quality control was performed, which would not hinder the evaluation of long-term trend in FVC. Thirdly, although FVC is an objective measure, it may be influenced by subjective factors, like degree of forceful exhalation and posture. However, CNSSCH technicians were well trained and followed a uniform standard, and acceptable range of instrument errors ≤ 3%. Finally, use of regional-level exposure data in our study may introduce ecological bias, and effects of environmental factors on children’s FVC need further study based on individual exposure data.

## Conclusion

In summary, last 35 years have witnessed a declining trend in the APFV in China. FVC changes followed a “V-shaped” trend, hitting bottom in 2005. Male had larger FVC than female, and urban students showed superior FVC than rural peers. FVC heterogeneity in 30 Chinese provinces exhibited four scenarios, and most minorities had lower FVC levels compared to Han. This study underscores the necessary of effective government policies in rural area, less developed provinces, and ethnic autonomous areas to enhance physical activity, alleviate academic pressure, and improve air quality and nutritional status. Further large-scale cohort studies are necessary to verify long-term trend in student’s lung function. Establishment of ethnicity-specific reference equation for lung function is urgent and important in China.

### Supplementary Information

**Additional file 1: Table S1.** Sex-specific number of Chinese Han students aged 7–22 years old in the CNSSCH, from 1985 to 2019. **Table S2.** Sex-specific number of Chinese minority students aged 7–18 years old in the CNSSCH 2019. **Table S3.** Types of spirometers used in different surveys. **Table S4.** Instructions for different spirometers. **Table S5.** Comparison of APFV and PFV among different groups of Chinese Han students. **Table S6.** Comparison of annual forced vital capacity (mL) changes of Chinese Han students in different age groups, from 1985 to 2019. **Table S7.** Difference of forced vital capacity in Chinese Han students from 1985 to 2019, by sex and region (urban or rural). **Table S8.** Forced vital capacity (mL) of Han students in all provinces of CNSSCH 2019. **Table S9.** Forced vital capacity (mL) of 27 nationalities students in CNSSCH 2019. **Table S10.** Spearman correlations between influencing factors and FVC level in 2019. **Figure S1.** Trend in FVC in Chinese Han students from 1985 to 2019, by sex, region, and age groups. **Figure S2.** Ranking of FVC of Chinese Han students in 30 provinces in 2019, by sex and region. **Figure S3.** Ranking of FVC of Chinese students of 27 nationalities in 2019, by sex. **Figure S4.** Relationships of influencing factors with Chinese male students' FVC in 2019 using GAM. **Figure S5.** Relationships of influencing factors with Chinese female students' FVC in 2019 using GAM.

## Data Availability

The data are available published reports about Chinese National Survey on Students’ Constitution and Health in 1985, 1991, 1995, 2000, 2005, 2010, 2014, and 2019. Longitude and latitude information of each province (municipality) are available in China National Geographic Information Center, (https://www.ngcc.cn/ngcc/html/1/54/75/index.html). Park green areas and urbanization rate of 2019 are available in the Chinese Statistical Yearbook 2020, (http://www.stats.gov.cn/sj/ndsj/2020/indexch.htm). Gross Domestic Product per capita of 2019 are available in the website of the National Bureau of Statistics of China, (https://data.stats.gov.cn/index.htm). The annual China regional-level mean particulate matter with aerodynamics diameter < 2.5 μm (PM_2.5_) data are available in the Atmospheric Composition Analysis Group, Washington University in St. Louis, USA (https://sites.wustl.edu/acag/datasets/surface-pm2-5/).

## Abbreviations

APFV: Age at peak forced vital capacity velocity
CNSSCH: Chinese National Survey on Students’ Constitution and Health
FVC: Forced vital capacity
PFV: Peak forced vital capacity velocity
SITAR: Super Imposition by Translation and Rotation
GDP: Gross domestic product
PM_2.5_: Particulate matter with aerodynamics diameter < 2.5 μm
GAM: Generalized additive model
