# Supplementary material for: Trend and heterogeneity in forced vital capacity among Chinese students during 1985–2019: results from Chinese National Survey on Students’ Constitution and Health
Source: Respir Res. 2023 Nov 6;24:268. doi: 10.1186/s12931-023-02573-5 (PMC10626663; doi:10.1186/s12931-023-02573-5)
Supplement: Supplementary file 1 — Additional file 1: Table S1. Sex-specific number of Chinese Han students aged 7–22 years old in the CNSSCH, from 1985 to 2019. Table S2. Sex-specific number of Chinese minority students aged 7–18 years old in the CNSSCH 2019. Table S3. Types of spirometers used in different surveys. Table S4. Instructions for different spirometers. Table S5. Comparison of APFV and PFV among different groups of Chinese Han students. Table S6. Comparison of annual forced vital capacity (mL) changes of Chinese Han students in different age groups, from 1985 to 2019. Table S7. Difference of forced vital capacity in Chinese Han students from 1985 to 2019, by sex and region (urban or rural). Table S8. Forced vital capacity (mL) of Han students in all provinces of CNSSCH 2019. Table S9. Forced vital capacity (mL) of 27 nationalities students in CNSSCH 2019. Table S10. Spearman correlations between influencing factors and FVC level in 2019. Figure S1. Trend in FVC in Chinese Han students from 1985 to 2019, by sex, region, and age groups. Figure S2. Ranking of FVC of Chinese Han students in 30 provinces in 2019, by sex and region. Figure S3. Ranking of FVC of Chinese students of 27 nationalities in 2019, by sex. Figure S4. Relationships of influencing factors with Chinese male students' FVC in 2019 using GAM. Figure S5. Relationships of influencing factors with Chinese female students' FVC in 2019 using GAM. [file 12931_2023_2573_MOESM1_ESM.docx]

**Additional material**

**Trend and heterogeneity in forced vital capacity among Chinese students during 1985-2019: Results from Chinese National Survey on Students’ Constitution and Health**

Siying Zhang, Lihong Wu, Yumei Zhong, Meirou Shao, Zhiyi Wei, Wenfeng Dong, Aiping Zhu, Fang-biao Tao, Xiulong Wu

**Table of Contents**

**Table S1:** Sex-specific number of Chinese Han students aged 7–22 years old in the CNSSCH, from 1985 to 2019.

**Table S2:** Sex-specific number of Chinese minority students aged 7–18 years old in the CNSSCH 2019.

**Table S3**: Types of spirometers used in different surveys.

**Table S4**: Instructions for different spirometers.

**Table S5**: Comparison of APFV and PFV among different groups of Chinese Han students.

**Table S6**: Comparison of annual forced vital capacity (mL) changes of Chinese Han students in different age groups, from 1985 to 2019.

**Table S7**: Difference of forced vital capacity in Chinese Han students from 1985 to 2019, by sex and region (urban or rural).

**Table S8**: Forced vital capacity (mL) of Han students in all provinces of CNSSCH 2019.

**Table S9**: Forced vital capacity (mL) of 27 nationalities students in CNSSCH 2019.

**Table S10**: Spearman correlations between influencing factors and FVC level in 2019.

**Figure S1**: Trend in FVC in Chinese Han students from 1985 to 2019, by sex, region, and age groups.

**Figure S2**: Ranking of FVC of Chinese Han students in 30 provinces in 2019, by sex

and region.

**Figure S3**: Ranking of FVC of Chinese students of 27 nationalities in 2019, by sex.

**Figure S4**: Relationships of influencing factors with Chinese male students' FVC in 2019 using GAM.

**Figure S5**: Relationships of influencing factors with Chinese female students' FVC in 2019 using GAM.

| Table S1: Sex-specific number of Chinese Han students aged 7–22 years old in the CNSSCH, from 1985 to 2019. | | | | | | | | |
| --- | --- | --- | --- | --- | --- | --- | --- | --- |
| Age (years) | 1985 | 1991 | 1995 | 2000 | 2005 | 2010 | 2014 | 2019 |
| **Male** | 237,476 | 93,597 | 127,423 | 133,725 | 145,842 | 131,428 | 130,893 | 128,293 |
| 7 | 17,106 | 5,817 | 8,747 | 9,052 | 9,627 | 8,969 | 8,943 | 9,081 |
| 8 | 17,119 | 5,949 | 8,740 | 9,082 | 9,629 | 8,967 | 8,928 | 9,040 |
| 9 | 17,113 | 5,931 | 8,742 | 9,026 | 9,730 | 8,968 | 8,966 | 9,078 |
| 10 | 17,117 | 5,826 | 8,745 | 9,124 | 9,847 | 8,980 | 8,977 | 8,907 |
| 11 | 17,122 | 5,884 | 8,750 | 9,007 | 9,850 | 8,984 | 8,983 | 8,952 |
| 12 | 17,119 | 5,868 | 8,736 | 9,024 | 9,709 | 8,978 | 8,946 | 8,930 |
| 13 | 17,115 | 5,826 | 8,738 | 8,905 | 9,670 | 8,976 | 8,968 | 8,849 |
| 14 | 17,119 | 5,867 | 8,749 | 9,008 | 9,664 | 8,984 | 8,973 | 8,886 |
| 15 | 17,115 | 5,914 | 8,745 | 9,040 | 9,928 | 8,979 | 8,969 | 8,901 |
| 16 | 17,116 | 5,895 | 8,682 | 9,017 | 9,801 | 8,962 | 8,962 | 8,841 |
| 17 | 17,092 | 5,898 | 8,729 | 8,899 | 9,786 | 8,952 | 8,977 | 8,783 |
| 18 | 16,847 | 5,933 | 8,654 | 9,095 | 10,117 | 8,963 | 8,579 | 8,407 |
| 19 | 8,415 | 5,798 | 5,608 | 6,573 | 7,433 | 5,966 | 5,953 | 5,479 |
| 20 | 8,493 | 5,944 | 5,678 | 6,474 | 7,348 | 5,973 | 5,950 | 5,671 |
| 21 | 8,462 | 5,549 | 5,704 | 6,640 | 7,191 | 5,965 | 5,972 | 5,543 |
| 22 | 7,006 | 5,698 | 5,676 | 5,759 | 6,512 | 5,862 | 5,847 | 4,945 |
| **Female** | 239,639 | 91,168 | 126,661 | 132,490 | 144,850 | 131,393 | 130,913 | 127,739 |
| 7 | 17,097 | 5,812 | 8,746 | 8,976 | 9,397 | 8,963 | 8,926 | 8,943 |
| 8 | 17,121 | 5,888 | 8,761 | 8,999 | 9,497 | 8,955 | 8,922 | 8,878 |
| 9 | 17,122 | 5,855 | 8,735 | 9,006 | 9,573 | 8,982 | 8,973 | 8,857 |
| 10 | 17,120 | 5,808 | 8,758 | 9,155 | 9,723 | 8,977 | 8,962 | 9,088 |
| 11 | 17,118 | 5,871 | 8,759 | 8,981 | 9,679 | 8,992 | 8,931 | 9,046 |
| 12 | 17,119 | 5,796 | 8,745 | 9,027 | 9,516 | 8,983 | 8,948 | 8,998 |
| 13 | 17,115 | 5,851 | 8,688 | 9,034 | 9,736 | 8,969 | 8,978 | 8,876 |
| 14 | 17,117 | 5,856 | 8,715 | 8,993 | 9,619 | 8,975 | 8,967 | 8,854 |
| 15 | 17,114 | 5,850 | 8,728 | 8,978 | 9,819 | 8,977 | 8,986 | 8,929 |
| 16 | 17,114 | 5,845 | 8,661 | 9,009 | 9,745 | 8,938 | 8,967 | 8,713 |
| 17 | 17,093 | 5,826 | 8,672 | 8,982 | 9,718 | 8,971 | 8,980 | 8,723 |
| 18 | 16,596 | 5,789 | 8,591 | 9,186 | 10,185 | 8,923 | 8,545 | 8,072 |
| 19 | 14,259 | 5,400 | 5,478 | 6,603 | 7,632 | 5,973 | 5,993 | 5,561 |
| 20 | 8,249 | 5,653 | 5,646 | 6,357 | 7,450 | 5,975 | 5,984 | 5,707 |
| 21 | 7,815 | 5,198 | 5,624 | 6,039 | 7,052 | 5,978 | 5,986 | 5,565 |
| 22 | 4,470 | 4,870 | 5,354 | 5,165 | 6,509 | 5,862 | 5,865 | 4,929 |
| Notes: Data were number of people; CNSSCH, Chinese National Survey on Students Constitution and Health. | | | | | | | | |

| Table S2: Sex-specific number of Chinese minority students aged 7–18 years old in the CNSSCH 2019. | | | | | | | | | | | | |
| --- | --- | --- | --- | --- | --- | --- | --- | --- | --- | --- | --- | --- |
| Minority | 7 | 8 | 9 | 10 | 11 | 12 | 13 | 14 | 15 | 16 | 17 | 18 |
| **Male** | 3,808 | 3,858 | 3,950 | 3,886 | 3,823 | 3,933 | 3,820 | 3,780 | 3,716 | 3,623 | 3,477 | 3,426 |
| Bai | 111 | 110 | 106 | 115 | 120 | 114 | 111 | 105 | 127 | 110 | 111 | 109 |
| Buyi | 100 | 98 | 98 | 100 | 100 | 98 | 100 | 98 | 100 | 99 | 100 | 98 |
| Dai | 109 | 110 | 113 | 109 | 113 | 106 | 110 | 114 | 115 | 115 | 111 | 109 |
| Dong | 89 | 113 | 100 | 99 | 99 | 100 | 101 | 99 | 98 | 102 | 98 | 100 |
| Dongxiang | 101 | 98 | 99 | 96 | 102 | 199 | 100 | 101 | 99 | 94 | 100 | 83 |
| Hani | 110 | 107 | 117 | 109 | 112 | 111 | 109 | 110 | 104 | 114 | 109 | 105 |
| Hui | 380 | 419 | 508 | 445 | 429 | 386 | 332 | 324 | 266 | 198 | 196 | 177 |
| Kazak | 111 | 104 | 103 | 112 | 109 | 114 | 113 | 113 | 118 | 110 | 104 | 106 |
| Kirgiz | 88 | 104 | 109 | 103 | 103 | 86 | 109 | 88 | 107 | 110 | 92 | 89 |
| Korean | 185 | 181 | 197 | 199 | 187 | 204 | 200 | 174 | 192 | 166 | 160 | 108 |
| Li | 125 | 123 | 118 | 133 | 129 | 134 | 120 | 123 | 130 | 134 | 102 | 107 |
| Lisu | 109 | 111 | 126 | 112 | 128 | 116 | 110 | 116 | 115 | 111 | 112 | 111 |
| Miao | 100 | 101 | 95 | 104 | 99 | 99 | 101 | 99 | 98 | 99 | 102 | 100 |
| Mongolian | 250 | 249 | 273 | 245 | 228 | 225 | 328 | 349 | 338 | 249 | 260 | 334 |
| Naxi | 111 | 110 | 115 | 116 | 110 | 110 | 105 | 115 | 104 | 110 | 109 | 109 |
| Qiang | 117 | 118 | 118 | 119 | 120 | 123 | 113 | 119 | 124 | 116 | 117 | 102 |
| Salar | 104 | 191 | 111 | 102 | 89 | 96 | 72 | 108 | 61 | 121 | 64 | 82 |
| Shui | 90 | 106 | 87 | 99 | 98 | 98 | 99 | 98 | 97 | 102 | 97 | 99 |
| Tibetan | 434 | 377 | 383 | 433 | 406 | 455 | 415 | 420 | 407 | 440 | 434 | 411 |
| Tu | 80 | 93 | 111 | 121 | 91 | 119 | 164 | 76 | 81 | 136 | 125 | 70 |
| Tujia | 101 | 105 | 122 | 80 | 102 | 101 | 100 | 97 | 101 | 117 | 134 | 134 |
| Uygur | 243 | 202 | 218 | 207 | 211 | 233 | 213 | 221 | 225 | 210 | 213 | 203 |
| Wa | 114 | 115 | 114 | 117 | 116 | 106 | 113 | 118 | 118 | 106 | 114 | 116 |
| Yao | 111 | 91 | 89 | 110 | 95 | 97 | 87 | 92 | 84 | 66 | 64 | 83 |
| Yi | 103 | 108 | 114 | 107 | 106 | 117 | 101 | 107 | 117 | 95 | 109 | 108 |
| Zhuang | 232 | 214 | 206 | 194 | 221 | 186 | 194 | 196 | 190 | 193 | 140 | 173 |
| **Female** | 3,746 | 3,795 | 3,764 | 3,847 | 3,746 | 3,812 | 3,856 | 3,709 | 3,691 | 3,789 | 3,648 | 3,249 |
| Bai | 110 | 113 | 126 | 110 | 111 | 112 | 110 | 115 | 109 | 112 | 112 | 120 |
| Buyi | 99 | 99 | 98 | 97 | 99 | 100 | 99 | 100 | 99 | 103 | 96 | 97 |
| Dai | 110 | 110 | 107 | 114 | 109 | 110 | 111 | 110 | 107 | 111 | 116 | 113 |
| Dong | 101 | 100 | 88 | 113 | 99 | 99 | 100 | 101 | 94 | 101 | 101 | 95 |
| Dongxiang | 100 | 100 | 99 | 97 | 100 | 101 | 99 | 98 | 99 | 94 | 102 | 60 |
| Hani | 108 | 109 | 115 | 111 | 106 | 116 | 107 | 112 | 111 | 109 | 110 | 109 |
| Hui | 349 | 380 | 396 | 382 | 428 | 344 | 320 | 355 | 305 | 305 | 301 | 19 |
| Kazak | 112 | 113 | 109 | 112 | 108 | 111 | 106 | 106 | 107 | 115 | 103 | 107 |
| Kirgiz | 99 | 100 | 103 | 100 | 95 | 71 | 104 | 86 | 107 | 108 | 96 | 92 |
| Korean | 185 | 181 | 197 | 199 | 187 | 204 | 200 | 174 | 192 | 166 | 160 | 108 |
| Li | 124 | 123 | 117 | 142 | 122 | 130 | 130 | 132 | 127 | 127 | 120 | 111 |
| Lisu | 110 | 112 | 110 | 108 | 108 | 110 | 111 | 114 | 112 | 132 | 118 | 112 |
| Miao | 100 | 100 | 100 | 98 | 102 | 100 | 100 | 100 | 98 | 99 | 100 | 98 |
| Mongolian | 253 | 230 | 256 | 258 | 229 | 245 | 344 | 312 | 342 | 263 | 280 | 333 |
| Naxi | 112 | 112 | 105 | 106 | 112 | 109 | 110 | 111 | 112 | 109 | 112 | 105 |
| Qiang | 108 | 116 | 112 | 111 | 113 | 109 | 106 | 112 | 122 | 109 | 115 | 105 |
| Salar | 107 | 174 | 158 | 108 | 93 | 97 | 92 | 65 | 63 | 176 | 117 | 65 |
| Shui | 100 | 97 | 100 | 91 | 100 | 99 | 101 | 98 | 100 | 97 | 100 | 98 |
| Tibetan | 427 | 391 | 339 | 456 | 378 | 450 | 408 | 402 | 432 | 427 | 441 | 384 |
| Tu | 64 | 92 | 92 | 76 | 81 | 131 | 148 | 64 | 55 | 121 | 134 | 81 |
| Tujia | 99 | 100 | 115 | 112 | 100 | 99 | 126 | 100 | 95 | 116 | 102 | 107 |
| Uygur | 233 | 209 | 221 | 214 | 215 | 238 | 185 | 232 | 208 | 201 | 182 | 230 |
| Wa | 108 | 106 | 112 | 113 | 121 | 113 | 112 | 116 | 141 | 111 | 129 | 115 |
| Yao | 102 | 92 | 78 | 105 | 104 | 102 | 117 | 90 | 86 | 71 | 63 | 97 |
| Yi | 105 | 107 | 112 | 116 | 98 | 116 | 108 | 104 | 107 | 112 | 104 | 102 |
| Zhuang | 221 | 229 | 199 | 198 | 228 | 196 | 202 | 200 | 161 | 194 | 134 | 186 |
| Notes: Data were number of people; CNSSCH, Chinese National Survey on Students Constitution and Health; 7-22 in the first row of the table represented age. | | | | | | | | | | | | |

| Table S3: Types of spirometers used in different surveys. | |
| --- | --- |
| Year | Equipment |
| 1985 | Rotary spirometer |
| 1991 | Rotary spirometer |
| 1995 | Electronic spirometer/needle spirometer/rotary spirometer |
| 2000 | Electronic spirometer/rotary spirometer |
| 2005 | Electronic spirometer |
| 2010 | Electronic spirometer |
| 2014 | Electronic spirometer |
| 2019 | Electronic spirometer |

| Table S4: Instructions for different spirometers. | |
| --- | --- |
| Instrumentation | Methods |
| Rotary spirometer | User should firstly adjust the water level in the cylinder to ensure it is flush with the lower edge of the water level line. Note that the handle of the blowing valve is deflated, and participant can blow when it is turned to one side. After participant has exhaled, the tester reads the spirometry value according to the position indicated by the vernier when the rotary cylinder stops, and the reading is accurate to 20 mL. |
| Needle spirometer | Check the sealing of rubber gasket, then adjust the knob and return the pointer to zero. Participant mouths to mouthpiece and blows, when blowing is complete, the value shown on the instrument dial is the value of lung capacity. If the value is abnormal, tester should check whether the connection between metal hose and air inlet is leaking. |
| Electronic spirometer | Place on a smooth table and perform a standard gas volume test before use. Spirometer test should be carried out in a well-ventilated room. Turn on the switch, and when the flashing signal on the display is fixed at "0", it indicates that the spirometer has entered the working state. Before the test, the tester should first put the mouthpiece on the air inlet and give it to the participant; explain the key points to the participant and tell him not to be nervous. Let participant try his best to inhale deeply until he can no longer inhale, and then exhale quickly and deeply. |

| Table S5: Comparison of APFV and PFV among different groups of Chinese Han students. | | | | | |
| --- | --- | --- | --- | --- | --- |
| Year | Urban | |  | Rural | |
|  | APFV (years) | PFV (mL/year) |  | APFV (years) | PFV (mL/year) |
| **Male** |  |  |  |  |  |
| 1985 | 13.85 ± 0.42 | 413.40 ± 78.21 |  | 13.75 ± 0.94 | 297.40 ± 41.98 |
| 1991 | 13.30 ± 1.20^*^ | 366.77 ± 37.71^*^ |  | 13.92 ± 0.25^*^ | 346.75 ± 20.92^*^ |
| 1995 | 13.10 ± 1.01^*^ | 376.22 ± 98.67^*^ |  | 13.63 ± 0.63^*^ | 320.76 ± 43.88^*^ |
| 2000 | 13.45 ± 0.18^*^ | 363.41 ± 76.53^*^ |  | 13.90 ± 0.10^*^ | 374.77 ± 11.39^*^ |
| 2005 | 13.38 ± 0.18^*^ | 358.68 ± 27.66^*^ |  | 13.78 ± 0.55^*^ | 336.61 ± 48.16^*^ |
| 2010 | 13.53 ± 0.46^*^ | 377.95 ± 26.46^*^ |  | 13.53 ± 0.38^*^ | 330.07 ± 78.88^*^ |
| 2014 | 13.13 ± 0.14^*^ | 396.40 ± 12.11^*^ |  | 13.15 ± 0.13^*^ | 366.05 ± 10.70^*^ |
| 2019 | 12.76 ± 0.13^*^ | 433.98 ± 30.73 |  | 13.01 ± 0.34^*^ | 384.97 ± 27.13^*^ |
| **Female** |  |  |  |  |  |
| 1985 | 13.35 ± 1.71 | 277.19 ± 26.39 |  | 11.68 ± 0.95 | 224.58 ± 19.68 |
| 1991 | 10.53 ± 0.75^*^ | 233.10 ± 26.29^*^ |  | 11.01 ± 0.61^*^ | 211.69 ± 42.17^*^ |
| 1995 | 10.68 ± 3.73^*^ | 201.28 ± 68.90^*^ |  | 10.84 ± 1.28^*^ | 214.16 ± 28.76^*^ |
| 2000 | 10.26 ± 1.01^*^ | 203.48 ± 26.43^*^ |  | 11.04 ± 0.38^*^ | 191.06 ± 11.88^*^ |
| 2005 | 9.69 ± 0.72^*^ | 178.34 ± 12.80^*^ |  | 10.68 ± 1.95^*^ | 168.2 ± 10.04^*^ |
| 2010 | 8.78 ± 2.07^*^ | 178.76 ± 6.32^*^ |  | 10.16 ± 4.02^*^ | 178.98 ± 55.3^*^ |
| 2014 | 9.94 ± 1.00^*^ | 208.11 ± 6.74^*^ |  | 10.57 ± 0.94^*^ | 211.17 ± 18.9^*^ |
| 2019 | 10.18 ± 0.69^*^ | 239.89 ± 10.64^*^ |  | 9.81 ± 0.78^*^ | 208.39 ± 7.15^*^ |
| Notes: Data were presented as mean ± SD; APFV, age at peak forced vital capacity velocity; PFV, peak forced vital capacity velocity; *, represented *P* value <0.001 when compared with 1985. | | | | | |

| Table S6: Comparison of annual forced vital capacity (mL) changes of Chinese Han students in different age groups, from 1985 to 2019. | | | | | | | | | | | |
| --- | --- | --- | --- | --- | --- | --- | --- | --- | --- | --- | --- |
| Age (years) | 1985 | 2005 | 2005–1985 | |  | 2019 | 2019–2005 | |  | 2019–1985 | |
|  |  |  | Absolute increment | Increase rate |  |  | Absolute increment | Increase rate |  | Absolute increment | Increase rate |
| **Urban male** |  |  |  |  |  |  |  |  |  |  |  |
| 7-9 | 1,558.31 | 1,267.64 | −290.67 | −18.65% |  | 1,467.28 | 199.64 | 15.75% |  | −91.03 | −5.84% |
| 10-12 | 2,073.67 | 1,856.12 | −217.55 | −10.49% |  | 2,235.21 | 379.09 | 20.42% |  | 161.54 | 7.79% |
| 13-15 | 3,065.97 | 2,814.00 | −251.97 | −8.22% |  | 3,378.87 | 564.87 | 20.07% |  | 312.90 | 10.21% |
| 16-18 | 3,950.67 | 3,514.75 | −435.92 | −11.03% |  | 3,942.79 | 428.04 | 12.18% |  | −7.88 | −0.20% |
| 19-22 | 4,250.05 | 3,788.98 | −461.07 | −10.85% |  | 4,117.60 | 328.62 | 8.67% |  | −132.45 | −3.12% |
| **Urban female** |  |  |  |  |  |  |  |  |  |  |  |
| 7-9 | 1,414.35 | 1,135.38 | −278.97 | −19.72% |  | 1,343.62 | 208.24 | 18.34% |  | −70.73 | −5.00% |
| 10-12 | 1,965.32 | 1,643.37 | −321.95 | −16.38% |  | 2,012.24 | 368.87 | 22.45% |  | 46.92 | 2.39% |
| 13-15 | 2,543.33 | 2,064.22 | −479.11 | −18.84% |  | 2,515.24 | 451.02 | 21.85% |  | −28.09 | −1.10% |
| 16-18 | 2,794.82 | 2,299.35 | −495.47 | −17.73% |  | 2,672.79 | 373.44 | 16.24% |  | −122.03 | −4.37% |
| 19-22 | 2,956.00 | 2,489.04 | −466.96 | −15.80% |  | 2,742.70 | 253.66 | 10.19% |  | −213.30 | −7.22% |
| **Rural male** |  |  |  |  |  |  |  |  |  |  |  |
| 7-9 | 1,448.39 | 1,174.88 | −273.51 | −18.88% |  | 1,394.95 | 220.07 | 18.73% |  | −53.44 | −3.69% |
| 10-12 | 1,945.34 | 1,719.07 | −226.27 | −11.63% |  | 2,114.83 | 395.76 | 23.02% |  | 169.49 | 8.71% |
| 13-15 | 2,857.69 | 2,589.58 | −268.11 | −9.38% |  | 3,153.67 | 564.09 | 21.78% |  | 295.98 | 10.36% |
| 16-18 | 3,821.11 | 3,346.40 | −474.71 | −12.42% |  | 3,792.30 | 445.90 | 13.32% |  | −28.81 | −0.75% |
| 19-22 | 4,165.00 | 3,663.46 | −501.54 | −12.04% |  | 4,007.70 | 344.24 | 9.40% |  | −157.30 | −3.78% |
| **Rural female** |  |  |  |  |  |  |  |  |  |  |  |
| 7-9 | 1,307.79 | 1,042.54 | −265.25 | −20.28% |  | 1,280.31 | 237.77 | 22.81% |  | −27.48 | −2.10% |
| 10-12 | 1,798.01 | 1,505.43 | −292.58 | −16.27% |  | 1,908.90 | 403.47 | 26.80% |  | 110.89 | 6.17% |
| 13-15 | 2,471.34 | 1,953.40 | −517.94 | −20.96% |  | 2,370.77 | 417.37 | 21.37% |  | −100.57 | −4.07% |
| 16-18 | 2,787.77 | 2,213.32 | −574.45 | −20.61% |  | 2,553.52 | 340.20 | 15.37% |  | −234.25 | −8.40% |
| 19-22 | 2,917.00 | 2,397.30 | −519.70 | −17.82% |  | 2,673.40 | 276.10 | 11.52% |  | −243.60 | −8.35% |

| Table S7: Difference of forced vital capacity in Chinese Han students from 1985 to 2019, by sex and region (urban or rural). | | | | | | | | |
| --- | --- | --- | --- | --- | --- | --- | --- | --- |
| Years | 1985 | 1991 | 1995 | 2000 | 2005 | 2010 | 2014 | 2019 |
| **Urban male-Urban female** |  |  |  |  |  |  |  |  |
| 7-9 | 143.97 | 148.69 | 145.41 | 146.68 | 132.26 | 130.53 | 150.21 | 123.66 |
| 10-12 | 108.36 | 133.42 | 162.61 | 181.14 | 212.76 | 228.67 | 224.64 | 222.97 |
| 13-15 | 522.64 | 652.55 | 656.31 | 697.84 | 749.78 | 760.77 | 817.30 | 863.63 |
| 16-18 | 1,155.85 | 1,165.40 | 1,104.42 | 1,177.44 | 1,215.40 | 1,209.82 | 1,275.58 | 1,270.00 |
| 19-22 | 1,294.05 | 1,333.00 | 1,233.90 | 1,222.20 | 1,299.94 | 1,335.64 | 1,387.79 | 1,374.90 |
| **Rural male-Rural female** |  |  |  |  |  |  |  |  |
| 7-9 | 140.60 | 144.69 | 140.32 | 141.61 | 132.34 | 124.07 | 136.48 | 114.64 |
| 10-12 | 147.33 | 156.10 | 159.31 | 179.41 | 213.64 | 190.48 | 210.20 | 205.93 |
| 13-15 | 386.36 | 509.74 | 538.35 | 584.43 | 636.18 | 678.63 | 744.24 | 782.89 |
| 16-18 | 1,033.34 | 1,107.24 | 1,045.63 | 1,104.67 | 1,133.09 | 1,178.53 | 1,235.00 | 1,238.78 |
| 19-22 | 1,248.00 | 1,298.31 | 1,182.60 | 1,202.39 | 1,266.16 | 1,328.73 | 1,330.78 | 1,334.30 |
| **Urban male-Rural male** |  |  |  |  |  |  |  |  |
| 7-9 | 106.56 | 127.34 | 93.60 | 120.54 | 92.84 | 94.80 | 79.67 | 63.31 |
| 10-12 | 167.31 | 215.50 | 164.50 | 175.32 | 137.94 | 119.64 | 144.40 | 103.34 |
| 13-15 | 71.99 | 139.12 | 139.20 | 156.04 | 110.82 | 116.69 | 133.93 | 144.47 |
| 16-18 | 7.05 | 64.17 | 65.48 | 113.51 | 86.03 | 110.15 | 130.50 | 119.27 |
| 19-22 | 39.00 | 56.01 | 51.90 | 105.48 | 91.74 | 69.58 | 57.68 | 69.30 |
| **Urban female-Rural female** |  |  |  |  |  |  |  |  |
| 7-9 | 109.92 | 131.34 | 98.68 | 125.61 | 92.76 | 101.26 | 93.40 | 72.33 |
| 10-12 | 128.33 | 192.81 | 167.81 | 177.05 | 137.06 | 157.83 | 158.84 | 120.38 |
| 13-15 | 208.28 | 281.94 | 257.16 | 269.44 | 224.42 | 198.83 | 206.98 | 225.20 |
| 16-18 | 129.56 | 122.33 | 124.27 | 186.27 | 168.35 | 141.44 | 171.08 | 150.49 |
| 19-22 | 85.05 | 90.70 | 103.20 | 125.29 | 125.52 | 76.49 | 114.69 | 109.90 |

| Table S8: Forced vital capacity (mL) of Han students in all provinces of CNSSCH 2019. | | | | | | | | | | | | | | | | |
| --- | --- | --- | --- | --- | --- | --- | --- | --- | --- | --- | --- | --- | --- | --- | --- | --- |
| Province | 7 | 8 | 9 | 10 | 11 | 12 | 13 | 14 | 15 | 16 | 17 | 18 | 19 | 20 | 21 | 22 |
| **Urban male** |  |  |  |  |  |  |  |  |  |  |  |  |  |  |  |  |
| China average | 1,238.60 | 1,469.40 | 1,692.10 | 1,918.40 | 2,224.00 | 2,554.90 | 3,037.20 | 3,417.10 | 3,685.70 | 3,875.40 | 3,963.40 | 3,991.70 | 4,102.00 | 4,073.10 | 4,144.10 | 4,156.40 |
| Anhui | 1,217.90 | 1,524.50 | 1,601.00 | 1,856.60 | 2,163.00 | 2,500.70 | 2,932.50 | 3,481.60 | 3,978.10 | 4,123.60 | 4,232.20 | 4,152.70 | 4,185.00 | 4,008.70 | 4,279.30 | 4,054.90 |
| Beijing | 1,292.20 | 1,530.60 | 1,834.20 | 2,009.50 | 2,358.80 | 2,779.70 | 3,313.90 | 3,693.50 | 3,911.00 | 4,032.50 | 4,169.00 | 4,272.20 | 4,374.90 | 4,408.10 | 4,579.40 | 4,397.00 |
| Fujian | 1,217.90 | 1,524.50 | 1,601.00 | 1,856.60 | 2,163.00 | 2,500.70 | 2,932.50 | 3,481.60 | 3,978.10 | 4,123.60 | 4,232.20 | 4,152.70 | 4,185.00 | 4,008.70 | 4,279.30 | 4,054.90 |
| Gansu | 1,089.10 | 1,327.60 | 1,541.10 | 1,774.00 | 1,933.20 | 2,294.10 | 2,889.50 | 3,173.00 | 3,504.40 | 3,832.30 | 3,938.70 | 3,675.00 | 3,852.50 | 3,851.30 | 3,841.40 | 3,848.60 |
| Guangdong | 1,082.40 | 1,307.70 | 1,464.60 | 1,735.80 | 1,926.70 | 2,187.60 | 2,617.20 | 2,906.40 | 3,134.40 | 3,331.30 | 3,442.90 | 3,596.80 | 4,063.60 | 4,142.30 | 4,090.20 | 4,053.10 |
| Guangxi | 1,063.20 | 1,275.50 | 1,529.50 | 1,826.50 | 2,091.80 | 2,296.90 | 2,777.50 | 3,005.70 | 3,450.50 | 3,359.70 | 3,603.20 | 3,698.10 | 3,865.00 | 3,808.00 | 3,916.80 | 4,078.60 |
| Guizhou | 1,149.90 | 1,437.20 | 1,517.00 | 1,739.20 | 2,021.40 | 2,154.00 | 2,502.90 | 3,090.20 | 3,318.00 | 3,491.60 | 3,557.50 | 3,628.00 | 3,679.70 | 3,822.30 | 3,798.90 | 3,805.90 |
| Hainan | 1,010.00 | 1,174.40 | 1,401.00 | 1,601.80 | 1,907.40 | 2,163.50 | 2,499.40 | 2,844.50 | 2,984.70 | 3,300.30 | 3,263.10 | 3,203.70 | 3,481.60 | 3,386.90 | 3,749.20 | 3,911.90 |
| Hebei | 1,407.20 | 1,597.70 | 1,777.00 | 2,060.80 | 2,462.80 | 2,826.30 | 3,216.80 | 3,841.00 | 3,892.40 | 3,918.10 | 4,051.10 | 4,488.80 | 4,604.20 | 4,732.20 | 4,573.80 | 4,503.60 |
| Henan | 1,271.50 | 1,452.40 | 1,692.60 | 1,842.40 | 2,143.10 | 2,729.80 | 3,065.90 | 3,692.00 | 4,025.70 | 4,095.50 | 4,231.70 | 4,320.80 | 4,038.20 | 4,052.30 | 4,013.80 | 4,098.50 |
| Heilongjiang | 1,096.40 | 1,351.90 | 1,636.70 | 1,874.00 | 2,150.40 | 2,482.90 | 3,102.00 | 3,317.80 | 3,501.00 | 3,771.80 | 3,745.50 | 3,950.60 | 4,005.50 | 4,156.60 | 4,171.80 | 4,373.20 |
| Hubei | 1,375.20 | 1,594.00 | 1,769.50 | 1,982.20 | 2,322.60 | 2,847.50 | 3,186.00 | 3,638.50 | 3,988.10 | 4,256.30 | 4,191.90 | 4,009.60 | 4,008.70 | 4,008.60 | 4,044.10 | 4,181.90 |
| Hunan | 1,113.20 | 1,299.40 | 1,492.50 | 1,742.60 | 2,021.30 | 2,307.50 | 2,674.90 | 2,966.00 | 3,229.40 | 3,562.00 | 3,695.60 | 3,628.00 | 3,908.00 | 3,989.90 | 4,080.90 | 4,138.10 |
| Jilin | 1,241.20 | 1,472.50 | 1,663.10 | 2,090.60 | 2,351.00 | 2,848.60 | 3,293.80 | 3,779.30 | 3,938.60 | 3,826.40 | 3,852.10 | 4,080.30 | 4,711.50 | 4,555.90 | 4,549.30 | 4,499.80 |
| Jiangsu | 1,347.10 | 1,492.40 | 1,623.00 | 1,946.30 | 2,255.00 | 2,746.90 | 3,343.80 | 3,697.90 | 3,687.20 | 3,985.30 | 4,046.00 | 4,011.20 | 4,199.40 | 4,145.80 | 4,125.70 | 4,030.40 |
| Jiangxi | 1,518.00 | 1,710.60 | 2,014.80 | 2,212.90 | 2,520.20 | 2,911.30 | 3,422.10 | 3,710.00 | 4,051.70 | 4,299.20 | 4,357.50 | 3,822.80 | 4,039.50 | 4,066.40 | 4,051.80 | 3,919.60 |
| Liaoning | 1,312.60 | 1,419.00 | 1,865.90 | 2,072.90 | 2,288.10 | 2,670.80 | 3,328.20 | 3,628.60 | 3,877.80 | 4,059.50 | 4,092.30 | 4,209.10 | 3,968.30 | 3,855.70 | 3,948.90 | 3,843.30 |
| Inner Mongolia | 1,302.90 | 1,629.90 | 1,827.50 | 2,091.30 | 2,470.80 | 2,773.20 | 3,108.60 | 3,585.70 | 3,738.40 | 3,701.60 | 3,907.80 | 3,907.10 | 3,875.90 | 3,709.00 | 3,984.00 | 4,081.20 |
| Ningxia | 943.90 | 1,104.10 | 1,210.70 | 1,462.00 | 1,606.30 | 2,013.00 | 2,739.20 | 3,042.40 | 3,405.50 | 3,420.20 | 3,641.50 | 3,759.90 | 3,721.60 | 3,675.00 | 3,647.00 | 3,359.50 |
| Qinghai | 1,137.00 | 1,315.90 | 1,482.50 | 1,642.70 | 1,798.10 | 1,966.20 | 2,450.90 | 2,744.70 | 2,956.50 | 3,314.40 | 3,391.70 | 3,492.00 | NA | 3,874.50 | 3,761.70 | NA |
| Shandong | 1,339.20 | 1,634.20 | 1,884.00 | 2,153.90 | 2,629.50 | 3,222.00 | 3,604.70 | 3,963.80 | 4,444.50 | 4,365.20 | 4,409.60 | 4,678.90 | 4,793.20 | 4,686.70 | 4,792.10 | 4,760.70 |
| Shanxi | 1,298.10 | 1,569.80 | 1,801.50 | 2,019.90 | 2,272.00 | 2,453.60 | 2,979.80 | 3,364.40 | 3,688.20 | 3,968.80 | 4,130.00 | 4,146.30 | 4,013.30 | 4,064.60 | 4,224.70 | 4,158.60 |
| Shaanxi | 1,201.80 | 1,477.90 | 1,675.20 | 1,896.00 | 2,236.90 | 2,444.20 | 2,993.40 | 3,182.40 | 3,689.30 | 3,651.30 | 3,668.30 | 3,813.50 | 4,257.80 | 4,375.10 | 4,367.90 | 4,565.30 |
| Shanghai | 1,351.50 | 1,563.90 | 1,790.50 | 1,980.50 | 2,350.60 | 2,830.30 | 3,286.70 | 3,670.50 | 3,891.30 | 4,214.90 | 4,207.20 | 4,133.10 | 4,246.50 | 4,211.60 | 4,411.60 | 4,529.30 |
| Sichuan | 1,390.80 | 1,620.50 | 1,879.10 | 2,145.20 | 2,452.50 | 2,611.90 | 3,011.90 | 3,313.10 | 3,752.10 | 3,902.20 | 4,015.80 | 4,122.70 | 3,803.40 | 3,835.50 | 3,928.00 | 4,053.90 |
| Tianjin | 1,513.30 | 1,737.50 | 2,013.00 | 2,306.20 | 2,576.80 | 2,754.10 | 3,365.00 | 3,939.50 | 4,063.50 | 4,439.00 | 4,373.10 | 4,505.10 | 4,511.80 | 4,183.80 | 4,254.10 | 4,479.40 |
| Xinjiang | 1,352.20 | 1,631.60 | 1,778.70 | 1,860.40 | 2,256.80 | 2,677.50 | 3,284.70 | 3,589.80 | 3,911.90 | 4,213.90 | 4,452.50 | 4,421.20 | 4,059.80 | 4,033.20 | 4,011.70 | 4,000.20 |
| Yunnan | 1,055.30 | 1,316.00 | 1,565.50 | 1,706.80 | 1,937.20 | 2,213.50 | 2,618.60 | 2,975.90 | 3,150.10 | 3,488.60 | 3,671.10 | 3,532.90 | 3,743.30 | 3,778.00 | 3,703.80 | 3,623.20 |
| Zhejiang | 1,322.90 | 1,438.10 | 1,942.90 | 2,068.50 | 2,498.20 | 2,763.30 | 3,137.70 | 3,551.90 | 3,992.90 | 4,097.50 | 4,383.70 | 4,162.60 | 4,242.20 | 4,181.60 | 4,384.70 | 4,075.70 |
| Chongqing | 1,144.80 | 1,488.20 | 1,711.90 | 1,911.70 | 2,255.80 | 2,521.80 | 3,079.70 | 3,449.10 | 3,781.80 | 3,892.40 | 3,834.50 | 3,881.60 | 3,858.60 | 3,853.80 | 3,899.40 | 4,106.90 |
| **Urban female** |  |  |  |  |  |  |  |  |  |  |  |  |  |  |  |  |
| China average | 1,200.40 | 1,394.10 | 1,592.00 | 1,835.50 | 2,098.80 | 2,416.40 | 2,799.90 | 3,164.30 | 3,488.50 | 3,696.30 | 3,818.60 | 3,866.40 | 3,946.60 | 4,013.60 | 4,046.60 | 4,024.10 |
| Anhui | 1,097.50 | 1,403.80 | 1,495.90 | 1,694.60 | 1,999.30 | 2,124.50 | 2,336.00 | 2,516.10 | 2,634.70 | 2,799.10 | 2,711.40 | 2,722.80 | 2,461.20 | 2,755.90 | 2,822.00 | 2,777.60 |
| Beijing | 1,155.00 | 1,399.70 | 1,668.80 | 1,903.70 | 2,201.20 | 2,471.90 | 2,689.90 | 2,768.10 | 2,776.10 | 2,944.60 | 2,985.70 | 2,974.90 | 2,950.60 | 3,018.70 | 3,063.10 | 3,038.40 |
| Fujian | 1,097.50 | 1,403.80 | 1,495.90 | 1,694.60 | 1,999.30 | 2,124.50 | 2,336.00 | 2,516.10 | 2,634.70 | 2,799.10 | 2,711.40 | 2,722.80 | 2,461.20 | 2,755.90 | 2,822.00 | 2,777.60 |
| Gansu | 1,031.10 | 1,140.20 | 1,277.10 | 1,620.30 | 1,754.80 | 2,067.80 | 2,368.90 | 2,433.10 | 2,548.00 | 2,652.90 | 2,631.40 | 2,547.20 | 2,690.10 | 2,558.60 | 2,608.50 | 2,595.70 |
| Guangdong | 1,000.70 | 1,221.50 | 1,384.90 | 1,555.40 | 1,790.30 | 1,878.10 | 2,173.70 | 2,221.20 | 2,282.40 | 2,459.90 | 2,444.40 | 2,445.30 | 2,775.30 | 2,563.60 | 2,698.20 | 2,721.40 |
| Guangxi | 932.00 | 1,138.30 | 1,343.50 | 1,641.60 | 1,805.30 | 2,030.80 | 2,028.10 | 2,270.10 | 2,309.00 | 2,250.80 | 2,323.50 | 2,407.40 | 2,641.40 | 2,675.80 | 2,742.20 | 2,678.10 |
| Guizhou | 998.60 | 1,252.50 | 1,365.80 | 1,649.70 | 1,903.00 | 1,964.40 | 2,121.10 | 2,119.50 | 2,153.50 | 2,424.50 | 2,437.80 | 2,359.10 | 2,545.20 | 2,573.50 | 2,551.40 | 2,508.20 |
| Hainan | 929.40 | 1,123.80 | 1,292.00 | 1,485.50 | 1,738.20 | 1,813.00 | 1,896.50 | 1,942.50 | 2,070.80 | 2,167.40 | 2,136.70 | 2,116.70 | 2,439.70 | 2,342.50 | 2,623.00 | 2,509.70 |
| Hebei | 1,269.90 | 1,490.60 | 1,715.90 | 1,915.10 | 2,216.60 | 2,550.90 | 2,539.50 | 2,643.90 | 2,769.90 | 2,628.00 | 2,800.00 | 2,913.70 | 3,011.70 | 2,997.00 | 3,018.50 | 3,031.70 |
| Henan | 1,160.40 | 1,311.40 | 1,637.60 | 1,669.00 | 1,975.50 | 2,191.20 | 2,487.10 | 2,636.00 | 2,598.30 | 2,717.40 | 2,788.40 | 2,835.70 | 2,585.90 | 2,638.80 | 2,607.90 | 2,649.40 |
| Heilongjiang | 1,014.00 | 1,206.10 | 1,430.40 | 1,692.20 | 1,861.70 | 2,026.60 | 2,348.40 | 2,441.50 | 2,499.10 | 2,562.30 | 2,475.20 | 2,588.90 | 2,727.60 | 2,719.20 | 2,566.80 | 3,424.00 |
| Hubei | 1,275.80 | 1,432.30 | 1,559.40 | 1,875.60 | 2,038.50 | 2,394.90 | 2,587.20 | 2,641.40 | 2,755.00 | 2,713.90 | 2,861.50 | 2,630.80 | 2,667.00 | 2,733.20 | 2,805.10 | 2,819.20 |
| Hunan | 999.00 | 1,263.50 | 1,339.80 | 1,656.20 | 1,817.10 | 1,978.90 | 2,269.70 | 2,265.70 | 2,371.30 | 2,341.80 | 2,402.50 | 2,277.80 | 2,722.30 | 2,749.10 | 2,861.00 | 2,755.60 |
| Jilin | 1,145.90 | 1,369.70 | 1,518.30 | 1,895.30 | 2,128.60 | 2,459.40 | 2,679.40 | 2,667.60 | 2,707.80 | 2,675.50 | 2,662.70 | 2,538.00 | 2,706.40 | 2,786.80 | 2,757.20 | 2,884.80 |
| Jiangsu | 1,262.10 | 1,395.90 | 1,538.00 | 1,879.00 | 2,174.00 | 2,415.40 | 2,528.70 | 2,635.70 | 2,751.00 | 2,855.40 | 2,816.50 | 2,666.60 | 2,621.80 | 2,672.70 | 2,699.00 | 2,541.60 |
| Jiangxi | 1,384.70 | 1,596.90 | 1,904.80 | 2,058.60 | 2,183.90 | 2,573.00 | 2,710.30 | 2,949.30 | 2,984.50 | 3,111.10 | 3,063.00 | 2,659.40 | 2,759.50 | 2,771.20 | 2,764.50 | 2,627.70 |
| Liaoning | 1,178.20 | 1,275.20 | 1,754.40 | 1,969.10 | 1,963.00 | 2,337.20 | 2,635.40 | 2,719.70 | 2,582.20 | 2,704.80 | 2,757.60 | 2,759.60 | 2,531.50 | 2,499.30 | 2,592.20 | 2,554.60 |
| Inner Mongolia | 1,235.20 | 1,499.90 | 1,697.70 | 1,944.10 | 2,282.20 | 2,385.70 | 2,590.40 | 2,695.50 | 2,670.60 | 2,494.50 | 2,666.70 | 2,597.40 | 2,741.00 | 2,512.80 | 2,715.00 | 2,673.30 |
| Ningxia | 874.70 | 1,032.00 | 1,128.40 | 1,236.60 | 1,391.80 | 1,892.50 | 2,145.00 | 2,249.90 | 2,388.90 | 2,432.20 | 2,374.60 | 2,409.10 | 2,526.40 | 2,481.30 | 2,508.00 | 2,257.60 |
| Qinghai | 1,027.70 | 1,151.70 | 1,323.20 | 1,516.80 | 1,694.80 | 1,861.30 | 2,168.80 | 2,245.30 | 2,270.40 | 2,186.70 | 2,305.70 | 2,537.70 | 2,614.10 | 2,526.30 | 2,671.70 | 2,381.30 |
| Shandong | 1,198.00 | 1,454.80 | 1,621.40 | 2,057.40 | 2,396.50 | 2,536.90 | 2,813.30 | 2,858.10 | 3,091.40 | 3,134.20 | 3,042.60 | 3,162.20 | 3,236.50 | 3,192.20 | 3,228.90 | 3,206.70 |
| Shanxi | 1,201.90 | 1,440.70 | 1,590.90 | 1,875.80 | 2,137.90 | 2,269.90 | 2,404.10 | 2,552.60 | 2,676.10 | 2,767.80 | 2,825.40 | 2,728.00 | 2,743.00 | 2,827.60 | 2,816.30 | 2,762.60 |
| Shaanxi | 1,096.80 | 1,366.30 | 1,518.10 | 1,697.80 | 1,972.30 | 2,100.60 | 2,393.00 | 2,304.80 | 2,499.60 | 2,470.80 | 2,494.60 | 2,508.40 | 2,863.30 | 2,749.60 | 2,787.60 | 2,820.20 |
| Shanghai | 1,214.50 | 1,497.80 | 1,599.20 | 1,883.50 | 2,228.30 | 2,471.70 | 2,632.30 | 2,815.60 | 2,805.00 | 2,865.50 | 2,912.10 | 2,923.00 | 2,804.60 | 2,909.10 | 2,970.80 | 2,918.10 |
| Sichuan | 1,401.00 | 1,518.10 | 1,789.00 | 2,031.70 | 2,333.40 | 2,358.40 | 2,475.00 | 2,467.70 | 2,723.70 | 2,772.80 | 2,696.30 | 2,778.10 | 2,548.20 | 2,607.60 | 2,547.60 | 2,722.70 |
| Tianjin | 1,363.60 | 1,597.70 | 1,795.30 | 2,054.60 | 2,426.60 | 2,551.80 | 2,607.40 | 2,872.90 | 2,929.60 | 3,036.80 | 3,080.40 | 3,063.90 | 2,895.50 | 2,885.70 | 2,811.20 | 2,784.20 |
| Xinjiang | 1,244.50 | 1,507.60 | 1,571.90 | 1,767.30 | 2,079.10 | 2,390.80 | 2,727.10 | 2,692.20 | 2,777.20 | 2,852.40 | 2,924.40 | 2,857.00 | 2,647.90 | 2,725.10 | 2,536.70 | 2,659.70 |
| Yunnan | 975.20 | 1,189.40 | 1,336.10 | 1,513.00 | 1,715.20 | 1,855.50 | 2,014.30 | 2,130.20 | 2,242.00 | 2,393.00 | 2,342.90 | 2,345.40 | 2,496.50 | 2,586.40 | 2,504.70 | 2,382.80 |
| Zhejiang | 1,191.20 | 1,300.00 | 1,784.70 | 1,994.90 | 2,270.20 | 2,452.70 | 2,547.90 | 2,670.20 | 2,888.00 | 2,918.60 | 2,951.70 | 2,839.10 | 2,885.20 | 3,039.50 | 3,026.70 | 2,899.80 |
| Chongqing | 1,302.90 | 1,538.80 | 1,579.60 | 1,778.70 | 2,100.00 | 2,389.40 | 2,307.80 | 2,643.70 | 2,618.90 | 2,720.00 | 2,789.00 | 2,703.40 | 2,638.60 | 2,652.90 | 2,624.90 | 2,736.50 |
| **Rural male** |  |  |  |  |  |  |  |  |  |  |  |  |  |  |  |  |
| China average | 1,139.40 | 1,350.80 | 1,538.00 | 1,773.70 | 2,023.70 | 2,237.50 | 2,425.40 | 2,526.00 | 2,593.60 | 2,660.10 | 2,690.00 | 2,667.80 | 2,719.40 | 2,733.60 | 2,759.10 | 2,761.40 |
| Anhui | 1,229.90 | 1,473.20 | 1,713.60 | 1,940.30 | 2,255.10 | 2,504.00 | 2,856.70 | 3,418.70 | 3,754.50 | 3,995.40 | 4,060.20 | 4,062.70 | 3,858.40 | 4,235.50 | 4,376.00 | 4,124.80 |
| Beijing | 1,280.90 | 1,547.40 | 1,781.30 | 2,047.90 | 2,327.90 | 2,853.90 | 3,193.10 | 3,426.80 | 3,886.40 | 4,180.70 | 4,285.80 | 4,323.80 | 4,185.20 | 4,338.20 | 4,469.90 | 4,429.50 |
| Fujian | 1,229.90 | 1,473.20 | 1,713.60 | 1,940.30 | 2,255.10 | 2,504.00 | 2,856.70 | 3,418.70 | 3,754.50 | 3,995.40 | 4,060.20 | 4,062.70 | 3,858.40 | 4,235.50 | 4,376.00 | 4,124.80 |
| Gansu | 1,024.60 | 1,206.40 | 1,457.60 | 1,650.40 | 1,869.00 | 2,062.50 | 2,720.50 | 3,039.60 | 3,243.10 | 3,431.10 | 3,684.30 | 3,544.00 | 3,847.60 | 3,695.40 | 3,722.50 | 3,792.70 |
| Guangdong | 1,090.80 | 1,273.50 | 1,527.90 | 1,835.70 | 2,028.00 | 2,483.90 | 2,730.00 | 3,141.90 | 3,340.00 | 3,403.80 | 3,603.50 | 3,661.20 | 3,829.30 | 3,909.80 | 3,853.60 | 3,933.90 |
| Guangxi | 1,060.90 | 1,318.60 | 1,463.90 | 1,699.70 | 1,944.90 | 2,179.40 | 2,357.90 | 2,702.90 | 3,084.90 | 3,326.90 | 3,506.10 | 3,529.20 | 3,639.00 | 3,704.90 | 3,867.40 | 3,792.40 |
| Guizhou | 1,127.90 | 1,294.40 | 1,452.50 | 1,583.10 | 1,848.80 | 2,115.50 | 2,560.20 | 2,743.70 | 2,996.30 | 3,344.80 | 3,411.20 | 3,405.70 | 3,773.50 | 3,775.50 | 3,754.10 | 3,829.70 |
| Hainan | 1,021.30 | 1,223.80 | 1,365.60 | 1,673.60 | 1,801.40 | 2,162.30 | 2,406.90 | 2,658.50 | 2,993.60 | 3,013.80 | 3,036.90 | 2,953.90 | 3,359.20 | 3,384.70 | 3,471.90 | 3,772.40 |
| Hebei | 1,473.00 | 1,709.30 | 1,892.40 | 2,178.00 | 2,368.60 | 2,749.10 | 3,344.30 | 3,562.80 | 4,005.80 | 4,199.40 | 4,202.90 | 4,472.80 | 4,405.00 | 4,570.00 | 4,481.30 | 4,325.70 |
| Henan | 1,215.30 | 1,480.40 | 1,601.60 | 1,737.00 | 2,094.30 | 2,346.40 | 2,555.60 | 2,668.60 | 3,759.10 | 3,770.00 | 3,992.30 | 4,156.60 | 3,836.50 | 3,915.00 | 3,940.30 | 4,028.10 |
| Heilongjiang | 1,203.20 | 1,425.60 | 1,748.10 | 1,982.70 | 2,189.60 | 2,504.90 | 2,787.60 | 3,179.50 | 3,812.30 | 3,994.80 | 4,227.00 | 3,716.60 | 3,946.00 | 4,092.60 | 4,038.40 | 4,310.60 |
| Hubei | 1,101.30 | 1,307.80 | 1,320.40 | 1,641.80 | 1,988.90 | 2,295.70 | 2,658.60 | 3,167.10 | 3,490.00 | 3,610.80 | 3,688.40 | 3,894.50 | 3,855.60 | 3,861.50 | 3,921.10 | 4,061.80 |
| Hunan | 963.10 | 1,137.70 | 1,286.90 | 1,589.60 | 1,773.10 | 2,227.00 | 2,668.60 | 3,083.00 | 3,343.00 | 3,448.30 | 3,589.60 | 3,495.70 | 3,824.40 | 3,637.00 | 3,904.90 | 3,909.60 |
| Jilin | 1,321.30 | 1,551.70 | 1,821.70 | 2,028.40 | 2,357.00 | 2,427.60 | 2,810.00 | 3,256.10 | 3,526.60 | 3,492.40 | 3,682.70 | 3,990.50 | 4,379.00 | 4,459.70 | 4,575.80 | 4,204.70 |
| Jiangsu | 1,234.20 | 1,439.40 | 1,669.70 | 1,903.20 | 2,229.20 | 2,742.60 | 3,200.20 | 3,294.10 | 3,907.20 | 3,906.90 | 4,116.10 | 4,087.70 | 3,987.50 | 3,891.20 | 3,981.50 | 4,001.50 |
| Jiangxi | 1,159.90 | 1,199.20 | 1,400.50 | 1,845.20 | 1,943.90 | 2,130.40 | 2,369.90 | 2,695.50 | 3,130.80 | 3,617.50 | 3,684.50 | 3,691.40 | 3,901.00 | 3,983.40 | 4,109.30 | 3,894.40 |
| Liaoning | 1,153.60 | 1,436.10 | 1,671.70 | 1,955.30 | 2,177.00 | 2,597.30 | 3,118.60 | 3,500.70 | 3,812.40 | 3,892.40 | 3,972.60 | 4,100.10 | 3,701.10 | 3,691.90 | 3,841.90 | 3,693.20 |
| Inner Mongolia | 1,260.30 | 1,435.40 | 1,694.00 | 1,916.30 | 2,177.60 | 2,561.30 | 3,017.40 | 3,339.50 | 3,490.40 | 3,563.80 | 3,840.70 | 3,925.50 | 3,856.00 | 3,921.80 | 4,024.80 | 3,994.10 |
| Ningxia | 1,106.90 | 1,276.90 | 1,408.00 | 1,521.90 | 1,774.30 | 2,101.90 | 2,495.30 | 2,878.30 | 3,046.00 | 3,358.00 | 3,588.60 | 3,465.90 | 3,497.60 | 3,542.80 | 3,497.90 | 3,670.20 |
| Qinghai | 1,143.10 | 1,313.70 | 1,453.10 | 1,705.40 | 2,054.40 | 2,296.00 | 2,525.10 | 3,094.20 | 3,237.80 | 3,615.20 | 3,814.80 | 3,923.00 | NA | 3,664.00 | NA | NA |
| Shandong | 1,446.60 | 1,731.80 | 1,919.50 | 2,178.20 | 2,463.50 | 2,978.60 | 3,203.90 | 3,739.50 | 4,028.40 | 4,217.80 | 4,215.00 | 4,557.10 | 4,703.80 | 4,685.60 | 4,739.60 | 4,731.80 |
| Shanxi | 1,310.30 | 1,543.70 | 1,736.20 | 1,915.30 | 2,233.50 | 2,441.40 | 2,889.10 | 3,132.00 | 3,428.50 | 3,836.30 | 3,884.30 | 3,792.60 | 4,045.40 | 4,020.60 | 4,011.30 | 3,962.00 |
| Shaanxi | 1,116.00 | 1,307.70 | 1,490.90 | 1,641.10 | 2,000.90 | 2,166.80 | 2,726.80 | 3,148.20 | 3,482.60 | 3,730.10 | 3,854.10 | 3,888.50 | 4,101.20 | 4,256.70 | 4,513.60 | 4,094.40 |
| Shanghai | 1,379.00 | 1,600.90 | 1,850.50 | 2,120.10 | 2,412.90 | 2,815.70 | 3,256.20 | 3,626.60 | 3,906.50 | 4,165.40 | 4,144.90 | 4,135.90 | 4,276.90 | 4,439.20 | 4,361.40 | 4,329.70 |
| Sichuan | 1,263.00 | 1,408.90 | 1,548.20 | 1,722.00 | 2,083.60 | 2,313.30 | 2,796.30 | 3,197.10 | 3,450.20 | 3,688.90 | 3,705.50 | 3,615.40 | 3,668.70 | 3,762.70 | 3,825.70 | 4,026.60 |
| Tianjin | 1,090.40 | 1,202.40 | 1,398.70 | 1,683.20 | 1,855.20 | 2,106.10 | 2,461.80 | 2,774.10 | 2,926.90 | 3,470.70 | 3,454.90 | 3,746.80 | 4,338.50 | 4,284.70 | 4,177.30 | 4,083.60 |
| Xinjiang | 1,338.20 | 1,477.20 | 1,772.90 | 1,902.20 | 2,178.60 | 2,463.00 | 2,760.60 | 3,260.90 | 3,678.80 | 4,144.70 | 4,326.80 | 4,213.50 | 3,783.40 | 4,019.70 | 3,928.70 | 3,859.10 |
| Yunnan | 1,145.80 | 1,297.90 | 1,487.90 | 1,655.60 | 1,924.30 | 2,163.40 | 2,467.30 | 2,888.20 | 3,142.70 | 3,217.50 | 3,613.30 | 3,715.90 | 3,552.70 | 3,627.20 | 3,593.40 | 3,602.20 |
| Zhejiang | 1,440.30 | 1,669.50 | 1,832.40 | 2,264.70 | 2,604.60 | 3,189.80 | 3,458.80 | 3,996.40 | 3,955.30 | 4,032.40 | 3,929.10 | 4,075.20 | 4,122.00 | 4,260.80 | 4,109.70 | 4,136.70 |
| Chongqing | 1,041.30 | 1,207.90 | 1,443.30 | 1,625.00 | 1,799.30 | 2,002.50 | 2,483.50 | 2,806.50 | 3,057.50 | 3,367.50 | 3,600.90 | 3,524.20 | 3,909.10 | 3,923.00 | 3,806.60 | 3,814.80 |
| **Rural female** |  |  |  |  |  |  |  |  |  |  |  |  |  |  |  |  |
| China average | 1,101.20 | 1,265.90 | 1,480.00 | 1,698.00 | 1,921.00 | 2,113.40 | 2,276.00 | 2,372.00 | 2,464.00 | 2,512.20 | 2,552.80 | 2,598.00 | 2,658.80 | 2,669.70 | 2,689.70 | 2,675.40 |
| Anhui | 1,112.20 | 1,361.60 | 1,528.20 | 1,664.30 | 1,984.00 | 2,162.00 | 2,281.70 | 2,519.60 | 2,573.60 | 2,673.20 | 2,662.00 | 2,683.20 | 2,505.50 | 2,719.80 | 2,780.30 | 2,701.80 |
| Beijing | 1,234.60 | 1,408.80 | 1,603.60 | 1,875.80 | 2,143.60 | 2,391.80 | 2,527.40 | 2,619.20 | 2,743.60 | 2,835.60 | 2,934.00 | 2,963.80 | 2,917.60 | 2,985.00 | 3,027.90 | 2,966.10 |
| Fujian | 1,112.20 | 1,361.60 | 1,528.20 | 1,664.30 | 1,984.00 | 2,162.00 | 2,281.70 | 2,519.60 | 2,573.60 | 2,673.20 | 2,662.00 | 2,683.20 | 2,505.50 | 2,719.80 | 2,780.30 | 2,701.80 |
| Gansu | 895.30 | 1,061.00 | 1,257.30 | 1,427.20 | 1,620.10 | 1,896.90 | 2,235.80 | 2,265.80 | 2,435.20 | 2,491.60 | 2,402.20 | 2,404.50 | 2,500.90 | 2,466.30 | 2,477.40 | 2,462.80 |
| Guangdong | 1,010.70 | 1,174.80 | 1,376.10 | 1,674.80 | 1,829.60 | 2,203.70 | 2,305.30 | 2,374.70 | 2,455.10 | 2,507.80 | 2,415.80 | 2,508.50 | 2,320.10 | 2,574.70 | 2,443.80 | 2,510.70 |
| Guangxi | 996.60 | 1,143.10 | 1,325.80 | 1,568.60 | 1,683.40 | 1,879.60 | 1,956.90 | 1,947.10 | 2,139.20 | 2,086.20 | 2,233.30 | 2,259.60 | 2,596.30 | 2,569.70 | 2,589.10 | 2,629.10 |
| Guizhou | 1,028.90 | 1,161.10 | 1,372.00 | 1,569.80 | 1,683.00 | 1,786.10 | 2,033.80 | 2,115.20 | 2,148.60 | 2,324.60 | 2,326.10 | 2,300.90 | 2,479.80 | 2,518.20 | 2,459.40 | 2,500.10 |
| Hainan | 946.60 | 1,020.90 | 1,303.00 | 1,474.90 | 1,633.60 | 1,817.50 | 1,835.10 | 1,942.90 | 2,140.60 | 2,102.90 | 2,100.20 | 2,092.10 | 2,140.30 | 2,215.80 | 2,206.90 | 2,378.70 |
| Hebei | 1,468.20 | 1,574.90 | 1,759.50 | 2,089.20 | 2,379.50 | 2,583.70 | 2,794.50 | 2,861.70 | 2,752.40 | 2,716.70 | 2,767.90 | 2,963.90 | 3,054.10 | 3,033.30 | 3,045.50 | 2,934.50 |
| Henan | 1,066.70 | 1,291.70 | 1,421.50 | 1,590.80 | 1,819.40 | 1,979.00 | 2,127.90 | 2,023.30 | 2,450.80 | 2,633.00 | 2,612.70 | 2,591.70 | 2,587.50 | 2,619.60 | 2,529.10 | 2,604.50 |
| Heilongjiang | 1,078.10 | 1,426.20 | 1,613.30 | 1,736.60 | 1,978.90 | 2,068.10 | 2,305.50 | 2,398.80 | 2,531.50 | 2,706.30 | 2,812.20 | 2,914.70 | 2,564.60 | 2,551.30 | 2,612.00 | 2,932.40 |
| Hubei | 1,051.00 | 1,195.10 | 1,420.80 | 1,710.80 | 1,912.50 | 2,071.30 | 2,308.10 | 2,299.10 | 2,333.10 | 2,388.00 | 2,560.70 | 2,630.30 | 2,669.00 | 2,677.70 | 2,701.30 | 2,739.60 |
| Hunan | 847.50 | 1,024.20 | 1,307.50 | 1,471.80 | 1,663.80 | 2,127.70 | 2,197.50 | 2,283.70 | 2,233.70 | 2,306.00 | 2,352.70 | 2,543.80 | 2,679.00 | 2,700.40 | 2,677.10 | 2,776.30 |
| Jilin | 1,210.60 | 1,364.10 | 1,706.20 | 1,881.00 | 2,311.10 | 2,200.00 | 2,133.80 | 2,416.30 | 2,361.30 | 2,275.40 | 2,391.90 | 2,593.20 | 2,931.30 | 2,730.00 | 2,714.20 | 2,586.80 |
| Jiangsu | 1,132.50 | 1,320.40 | 1,576.80 | 1,891.60 | 2,039.70 | 2,378.40 | 2,495.40 | 2,584.80 | 2,720.50 | 2,792.10 | 2,811.00 | 2,678.00 | 2,572.10 | 2,661.20 | 2,636.90 | 2,693.10 |
| Jiangxi | 1,013.70 | 1,168.90 | 1,486.90 | 1,583.80 | 1,718.20 | 1,819.30 | 1,937.70 | 2,171.40 | 2,168.20 | 2,300.80 | 2,418.20 | 2,441.50 | 2,721.10 | 2,704.70 | 2,673.80 | 2,541.40 |
| Liaoning | 1,077.80 | 1,246.80 | 1,484.60 | 1,717.20 | 1,977.40 | 2,364.70 | 2,548.80 | 2,704.60 | 2,704.30 | 2,768.20 | 2,776.20 | 2,707.70 | 2,468.90 | 2,429.30 | 2,500.60 | 2,519.60 |
| Inner Mongolia | 1,184.20 | 1,377.10 | 1,582.20 | 1,818.50 | 2,058.20 | 2,218.80 | 2,420.20 | 2,485.30 | 2,495.50 | 2,507.60 | 2,545.80 | 2,547.00 | 2,600.20 | 2,617.80 | 2,588.90 | 2,654.80 |
| Ningxia | 1,030.50 | 1,127.50 | 1,300.70 | 1,471.90 | 1,586.60 | 1,872.10 | 2,096.20 | 2,138.80 | 2,168.90 | 2,269.90 | 2,439.80 | 2,397.80 | 2,387.40 | 2,294.80 | 2,328.30 | 2,264.30 |
| Qinghai | 1,079.60 | 1,326.50 | 1,379.40 | 1,525.10 | 1,692.20 | 1,895.10 | 2,087.60 | 2,325.60 | 2,449.60 | 2,402.80 | 2,412.70 | 2,527.80 | 2,548.40 | 2,645.10 | 2,690.60 | 2,421.20 |
| Shandong | 1,209.80 | 1,523.20 | 1,780.60 | 2,051.60 | 2,169.50 | 2,374.80 | 2,462.60 | 2,570.60 | 2,876.60 | 2,810.20 | 2,861.10 | 3,157.00 | 3,148.50 | 3,084.70 | 3,180.30 | 3,229.10 |
| Shanxi | 1,176.50 | 1,336.40 | 1,554.40 | 1,783.30 | 2,008.30 | 2,142.00 | 2,309.30 | 2,407.60 | 2,474.90 | 2,653.40 | 2,701.10 | 2,613.30 | 2,699.60 | 2,598.30 | 2,707.20 | 2,704.20 |
| Shaanxi | 1,009.40 | 1,201.00 | 1,348.50 | 1,559.40 | 1,790.40 | 1,968.10 | 2,150.00 | 2,271.30 | 2,435.50 | 2,426.00 | 2,430.80 | 2,514.20 | 2,726.50 | 2,712.80 | 2,868.50 | 2,806.60 |
| Shanghai | 1,234.10 | 1,449.30 | 1,682.20 | 1,895.60 | 2,268.30 | 2,460.50 | 2,618.80 | 2,720.30 | 2,808.30 | 2,940.30 | 2,934.20 | 2,826.50 | 2,954.30 | 3,097.40 | 3,088.10 | 3,102.10 |
| Sichuan | 1,169.50 | 1,284.70 | 1,482.00 | 1,731.70 | 2,008.70 | 2,138.50 | 2,360.90 | 2,362.60 | 2,498.70 | 2,537.70 | 2,584.60 | 2,551.50 | 2,502.50 | 2,496.00 | 2,533.20 | 2,580.70 |
| Tianjin | 1,103.60 | 1,136.40 | 1,296.70 | 1,497.10 | 1,718.70 | 1,790.60 | 1,761.50 | 1,945.00 | 2,061.00 | 2,286.30 | 2,135.00 | 2,372.10 | 2,792.40 | 2,837.80 | 2,956.10 | 2,869.40 |
| Xinjiang | 1,219.90 | 1,337.60 | 1,637.10 | 1,768.90 | 2,001.00 | 2,254.90 | 2,365.20 | 2,529.10 | 2,745.00 | 2,679.70 | 2,784.60 | 2,840.90 | 2,579.80 | 2,652.70 | 2,675.60 | 2,401.20 |
| Yunnan | 1,050.30 | 1,148.80 | 1,291.70 | 1,440.70 | 1,659.60 | 1,771.80 | 2,047.60 | 2,050.80 | 2,163.90 | 2,175.70 | 2,329.50 | 2,445.70 | 2,511.70 | 2,394.00 | 2,452.70 | 2,345.30 |
| Zhejiang | 1,290.90 | 1,470.50 | 1,760.60 | 2,099.30 | 2,440.40 | 2,548.40 | 2,750.30 | 2,879.60 | 2,804.30 | 2,721.90 | 2,727.10 | 2,846.90 | 2,877.00 | 2,916.30 | 2,891.40 | 2,843.20 |
| Chongqing | 906.40 | 1,011.70 | 1,372.10 | 1,520.40 | 1,815.30 | 1,989.00 | 2,132.30 | 2,248.60 | 2,280.30 | 2,360.30 | 2,568.40 | 2,325.80 | 2,560.30 | 2,716.20 | 2,559.50 | 2,648.50 |
| Notes: CNSSCH, Chinese National Survey on Students Constitution and Health; 7-22 in the first row of the table represented age. | | | | | | | | | | | | | | | | |

| Table S9: Forced vital capacity (mL) of 27 nationalities students in CNSSCH 2019. | | | | | | | | | | | | |
| --- | --- | --- | --- | --- | --- | --- | --- | --- | --- | --- | --- | --- |
| Nationalities | 7 | 8 | 9 | 10 | 11 | 12 | 13 | 14 | 15 | 16 | 17 | 18 |
| **Male** |  |  |  |  |  |  |  |  |  |  |  |  |
| Han | 1,219.50 | 1,432.10 | 1,642.40 | 1,876.60 | 2,162.10 | 2,486.80 | 2,920.10 | 3,293.50 | 3,586.50 | 3,786.40 | 3,892.40 | 3,929.60 |
| Bai | 898.10 | 1,108.70 | 1,216.00 | 1,330.10 | 1,578.10 | 1,813.80 | 2,289.40 | 2,577.70 | 2,599.20 | 3,153.50 | 3,102.10 | 3,225.60 |
| Buyi | 1,300.60 | 1,494.30 | 1,611.50 | 1,792.10 | 1,852.50 | 2,152.30 | 2,435.80 | 2,810.80 | 3,260.90 | 3,499.90 | 3,585.30 | 3,357.60 |
| Dai | 872.90 | 1,070.20 | 1,167.30 | 1,295.10 | 1,496.90 | 1,551.30 | 1,808.50 | 2,192.30 | 2,415.10 | 2,717.20 | 2,874.30 | 3,041.00 |
| Dong | 963.90 | 1,115.20 | 1,344.80 | 1,569.30 | 1,948.10 | 1,963.20 | 2,428.50 | 2,790.20 | 2,938.80 | 3,322.10 | 3,545.80 | 3,491.90 |
| Dongxiang | 1,115.80 | 1,268.50 | 1,395.50 | 1,603.40 | 1,678.30 | 1,830.40 | 2,194.50 | 2,242.60 | 2,469.30 | 3,106.60 | 3,169.20 | 3,250.70 |
| Hani | 1,071.70 | 1,278.80 | 1,440.10 | 1,584.60 | 1,716.20 | 1,934.90 | 2,087.70 | 2,474.40 | 2,823.30 | 3,099.90 | 3,143.90 | 3,116.70 |
| Hui | 969.70 | 1,145.10 | 1,282.50 | 1,492.10 | 1,705.60 | 1,945.00 | 2,439.10 | 2,746.90 | 3,071.10 | 3,413.30 | 3,523.40 | 3,655.70 |
| Kazak | 1,304.40 | 1,575.20 | 1,787.80 | 2,031.00 | 2,236.70 | 2,545.00 | 2,935.40 | 3,281.90 | 3,847.20 | 3,998.30 | 3,969.50 | 4,269.50 |
| Kirgiz | 1,010.80 | 1,221.70 | 1,346.30 | 1,551.40 | 1,621.50 | 1,788.30 | 2,173.60 | 2,541.60 | 2,753.60 | 3,547.90 | 3,409.80 | 3,735.80 |
| Korean | 1,232.80 | 1,464.70 | 1,697.90 | 1,908.30 | 2,201.10 | 2,479.60 | 2,918.50 | 3,392.80 | 3,308.70 | 3,554.40 | 3,789.80 | 3,739.50 |
| Li | 1,129.40 | 1,307.60 | 1,461.10 | 1,570.70 | 1,823.00 | 2,111.40 | 2,406.80 | 2,635.50 | 3,001.20 | 3,221.80 | 3,165.00 | 3,288.20 |
| Lisu | 1,107.70 | 1,346.00 | 1,471.40 | 1,527.10 | 1,736.90 | 1,930.90 | 2,280.90 | 2,553.70 | 2,990.40 | 2,817.90 | 3,016.90 | 3,124.90 |
| Miao | 1,254.30 | 1,467.70 | 1,568.10 | 1,676.80 | 2,236.40 | 2,687.60 | 2,745.20 | 2,991.40 | 3,374.10 | 3,537.30 | 3,665.20 | 3,790.30 |
| Mongolian | 1,431.60 | 1,608.60 | 1,976.20 | 2,257.00 | 2,466.90 | 2,650.80 | 2,878.90 | 3,322.50 | 3,578.40 | 3,756.00 | 3,877.50 | 3,852.40 |
| Naxi | 838.80 | 1,003.20 | 1,328.10 | 1,551.60 | 1,664.50 | 1,974.90 | 2,432.70 | 2,751.80 | 2,953.30 | 3,248.00 | 3,281.20 | 3,253.60 |
| Qiang | 1,071.80 | 1,341.30 | 1,537.40 | 1,751.90 | 1,957.20 | 2,099.20 | 2,512.60 | 2,937.20 | 3,060.50 | 3,525.50 | 3,625.80 | 3,844.10 |
| Salar | 872.70 | 1,050.70 | 1,225.20 | 1,345.40 | 1,509.70 | 1,882.00 | 2,373.80 | 2,589.40 | 2,491.70 | 3,213.40 | 3,452.60 | 3,436.00 |
| Shui | 1,027.30 | 1,221.20 | 1,276.60 | 1,454.90 | 1,622.70 | 1,790.40 | 2,459.30 | 2,694.40 | 2,778.80 | 3,192.40 | 3,228.40 | 3,428.30 |
| Tibetan | 1,008.00 | 1,107.20 | 1,248.30 | 1,502.80 | 1,640.50 | 1,901.10 | 2,370.70 | 2,584.60 | 2,844.80 | 3,245.90 | 3,294.00 | 3,245.30 |
| Tu | 1,148.30 | 1,238.00 | 1,286.90 | 1,343.50 | 1,595.80 | 1,760.40 | 2,053.60 | 2,505.10 | 3,091.60 | 3,461.60 | 3,564.60 | 3,615.10 |
| Tujia | 983.70 | 1,356.60 | 1,423.90 | 1,267.40 | 1,516.70 | 1,717.20 | 2,401.20 | 2,280.80 | 2,446.50 | 3,256.60 | 3,950.30 | 3,913.40 |
| Uygur | 1,146.30 | 1,406.00 | 1,613.30 | 1,852.90 | 1,910.20 | 2,113.60 | 2,531.50 | 2,988.50 | 3,336.70 | 3,932.30 | 4,029.60 | 4,189.30 |
| Wa | 1,127.10 | 1,338.30 | 1,483.90 | 1,624.50 | 1,746.20 | 1,977.30 | 2,245.90 | 2,447.90 | 2,920.20 | 3,107.10 | 3,200.70 | 3,183.30 |
| Yao | 1,060.90 | 1,227.80 | 1,310.40 | 1,412.00 | 1,650.00 | 1,735.90 | 1,991.30 | 2,459.70 | 2,598.20 | 2,988.70 | 3,081.20 | 3,317.70 |
| Yi | 872.30 | 1,073.30 | 1,277.40 | 1,450.80 | 1,586.30 | 1,722.40 | 1,789.30 | 2,119.60 | 2,404.50 | 2,809.20 | 2,480.40 | 2,933.40 |
| Zhuang | 1,039.40 | 1,282.40 | 1,522.90 | 1,692.40 | 1,988.70 | 2,357.90 | 2,820.10 | 3,117.60 | 3,388.40 | 3,456.20 | 3,459.60 | 3,598.10 |
| **Female** |  |  |  |  |  |  |  |  |  |  |  |  |
| Han | 1,120.10 | 1,308.40 | 1,509.40 | 1,735.30 | 1,972.30 | 2,175.60 | 2,351.10 | 2,450.10 | 2,529.30 | 2,585.70 | 2,621.00 | 2,631.90 |
| Bai | 865.30 | 1,005.40 | 1,116.20 | 1,316.30 | 1,458.70 | 1,541.70 | 1,856.60 | 1,837.40 | 1,871.40 | 2,032.30 | 1,973.90 | 2,079.30 |
| Buyi | 1,216.50 | 1,313.20 | 1,547.50 | 1,730.00 | 1,902.60 | 1,948.40 | 2,050.90 | 2,276.30 | 2,240.30 | 2,571.40 | 2,506.80 | 2,479.50 |
| Dai | 852.00 | 1,022.70 | 1,029.90 | 1,205.10 | 1,278.30 | 1,351.00 | 1,468.60 | 1,705.30 | 1,730.60 | 1,777.90 | 1,872.40 | 1,933.00 |
| Dong | 806.00 | 957.80 | 1,219.60 | 1,357.00 | 1,601.60 | 1,722.60 | 1,944.30 | 2,092.70 | 2,197.90 | 2,332.10 | 2,404.70 | 2,274.10 |
| Dongxiang | 997.30 | 1,155.70 | 1,281.90 | 1,386.10 | 1,525.20 | 1,676.10 | 1,887.50 | 1,892.00 | 1,965.60 | 2,173.90 | 2,194.90 | 2,152.70 |
| Hani | 1,033.80 | 1,149.10 | 1,232.70 | 1,393.80 | 1,648.70 | 1,756.30 | 1,802.30 | 1,873.20 | 2,125.60 | 2,154.00 | 2,166.50 | 2,236.80 |
| Hui | 891.30 | 26.30 | 1,130.40 | 1,311.30 | 1,489.20 | 1,851.70 | 1,956.60 | 2,062.30 | 2,198.00 | 2,293.50 | 2,331.20 | 2,306.40 |
| Kazak | 1,266.70 | 1,419.70 | 1,592.50 | 1,775.70 | 2,034.40 | 2,061.50 | 2,373.90 | 2,542.70 | 2,785.80 | 2,539.10 | 2,627.90 | 2,616.10 |
| Kirgiz | 1,195.40 | 1,067.10 | 1,191.60 | 1,372.70 | 1,596.10 | 1,677.80 | 1,911.00 | 2,070.20 | 1,972.60 | 2,426.60 | 2,355.50 | 2,190.80 |
| Korean | 1,100.10 | 1,356.70 | 1,615.70 | 1,752.00 | 2,090.90 | 2,237.30 | 2,365.20 | 2,522.40 | 2,332.00 | 2,446.40 | 2,399.80 | 2,488.80 |
| Li | 876.90 | 1,013.50 | 1,268.20 | 1,336.90 | 1,486.80 | 1,762.40 | 1,842.40 | 2,034.00 | 2,022.20 | 2,140.20 | 2,188.40 | 2,140.50 |
| Lisu | 955.60 | 1,108.80 | 1,309.90 | 1,434.00 | 1,568.10 | 1,720.40 | 1,918.50 | 2,064.40 | 2,105.20 | 1,978.50 | 2,026.00 | 2,130.20 |
| Miao | 1,226.20 | 1,278.20 | 1,387.70 | 1,582.10 | 1,936.70 | 2,276.80 | 2,208.60 | 2,271.10 | 2,407.80 | 2,559.60 | 2,540.70 | 2,628.20 |
| Mongolian | 1,291.90 | 1,488.60 | 1,783.30 | 1,962.90 | 2,247.60 | 2,419.70 | 2,384.60 | 2,433.20 | 2,539.30 | 2,401.50 | 2,320.80 | 2,310.70 |
| Naxi | 771.90 | 901.20 | 1,127.00 | 1,388.90 | 1,545.30 | 1,736.90 | 1,855.80 | 1,972.80 | 2,092.40 | 1,960.80 | 2,089.90 | 2,084.00 |
| Qiang | 1,028.10 | 1,185.40 | 1,416.80 | 1,594.80 | 1,841.90 | 1,972.00 | 2,154.70 | 2,387.30 | 2,417.60 | 2,345.20 | 2,445.90 | 2,458.20 |
| Salar | 767.80 | 855.70 | 1,080.60 | 1,126.80 | 1,369.70 | 1,596.80 | 2,013.40 | 1,970.70 | 1,981.10 | 2,200.30 | 2,141.20 | 2,190.30 |
| Shui | 947.10 | 1,088.20 | 1,135.60 | 1,297.30 | 1,490.40 | 1,741.90 | 1,943.80 | 2,100.20 | 2,224.70 | 2,189.60 | 2,142.80 | 2,201.20 |
| Tibetan | 903.80 | 990.50 | 1,199.50 | 1,403.30 | 1,501.70 | 1,675.60 | 2,028.70 | 2,216.30 | 2,113.50 | 2,169.90 | 2,229.20 | 2,206.70 |
| Tu | 1,009.80 | 1,096.10 | 1,170.70 | 1,287.20 | 1,423.60 | 1,559.00 | 1,985.60 | 2,055.80 | 2,228.50 | 2,469.40 | 2,430.10 | 2,278.30 |
| Tujia | 910.30 | 1,226.10 | 1,371.90 | 1,343.20 | 1,413.60 | 1,459.90 | 1,945.80 | 1,929.50 | 1,941.70 | 2,229.80 | 2,447.80 | 2,426.10 |
| Uygur | 1,083.70 | 1,465.70 | 1,376.90 | 1,617.80 | 1,714.40 | 1,772.50 | 2,041.50 | 2,256.30 | 2,389.30 | 2,448.60 | 2,412.00 | 2,483.50 |
| Wa | 1,099.20 | 1,265.00 | 1,349.60 | 1,477.20 | 1,706.70 | 1,679.70 | 1,760.40 | 1,836.50 | 2,081.90 | 2,108.20 | 2,156.10 | 2,063.70 |
| Yao | 945.10 | 1,121.40 | 1,168.60 | 1,322.50 | 1,451.70 | 1,614.00 | 1,835.90 | 1,818.50 | 1,960.00 | 2,211.30 | 2,131.70 | 2,286.10 |
| Yi | 774.10 | 947.70 | 1,128.00 | 1,341.10 | 1,446.10 | 1,516.90 | 1,681.50 | 1,700.90 | 1,876.10 | 1,957.70 | 1,948.50 | 1,957.90 |
| Zhuang | 965.10 | 1,169.00 | 1,408.20 | 1,563.60 | 1,788.20 | 2,021.70 | 2,285.60 | 2,295.90 | 2,420.10 | 2,475.80 | 2,373.00 | 2,206.90 |
| Notes: CNSSCH, Chinese National Survey on Students Constitution and Health; 7-22 in the first row of the table represented age. | | | | | | | | | | | | |

| Table S10: Spearman correlations between influencing factors and FVC level in 2019. | | | | | | | | | | | |
| --- | --- | --- | --- | --- | --- | --- | --- | --- | --- | --- | --- |
|  | FVC | Age (year) | Sex | Area | Height (cm) | GDP per capita (thousand yuan/person) | Urbanization rate (%) | Longitude (degree) | Latitude (degree) | PM_2.5_ (μg/m^3^) | Park green space (km^2^) |
| FVC | **1.000** | 0.802 | −0.363 | −0.077 | 0.943 | 0.103 | 0.111 | 0.142 | 0.094 | 0.126 | 0.143 |
| Age (year) | <0.001 | **1.000** | 0.000 | 0.000 | 0.805 | 0.000 | 0.000 | 0.010 | 0.000 | 0.010 | 0.010 |
| Sex | <0.001 | 0.880 | **1.000** | 0.000 | −0.316 | 0.000 | 0.000 | 0.000 | 0.000 | 0.000 | 0.000 |
| Area | 0.001 | 0.974 | 0.982 | **1.000** | −0.049 | 0.000 | 0.000 | 0.000 | 0.000 | 0.000 | 0.000 |
| Height (cm) | <0.001 | <0.001 | <0.001 | 0.032 | **1.000** | 0.061 | 0.107 | 0.105 | 0.123 | 0.083 | 0.058 |
| GDP per capita (thousand yuan/person) | <0.001 | 0.909 | 0.931 | 0.986 | 0.008 | **1.000** | 0.788 | 0.392 | −0.119 | 0.259 | 0.481 |
| Urbanization rate (%) | <0.001 | 0.896 | 0.921 | 0.984 | <0.001 | <0.001 | **1.000** | 0.613 | 0.177 | 0.217 | 0.377 |
| Longitude (degree) | <0.001 | 0.813 | 0.858 | 0.972 | <0.001 | <0.001 | <0.001 | **1.000** | 0.243 | 0.281 | 0.538 |
| Latitude (degree) | <0.001 | 0.951 | 0.963 | 0.993 | <0.001 | <0.001 | <0.001 | <0.001 | **1.000** | 0.419 | 0.000 |
| PM_2.5_ (μg/m^3^) | <0.001 | 0.813 | 0.858 | 0.972 | <0.001 | <0.001 | <0.001 | <0.001 | <0.001 | **1.000** | 0.394 |
| Park green space (km^2^) | <0.001 | 0.800 | 0.848 | 0.969 | 0.011 | <0.001 | <0.001 | <0.001 | 0.947 | 0.000 | **1.000** |
| **Notes:** FVC, forced vital capacity; PM_2.5_, particulate matter with aerodynamics diameter <2.5 μm and the values in the table represented the average annual PM2.5 value in 2019. Right-upper part of table represented Spearman correlation coefficient, and left-lower part represented Spearman correlation *P*-value. | | | | | | | | | | | |


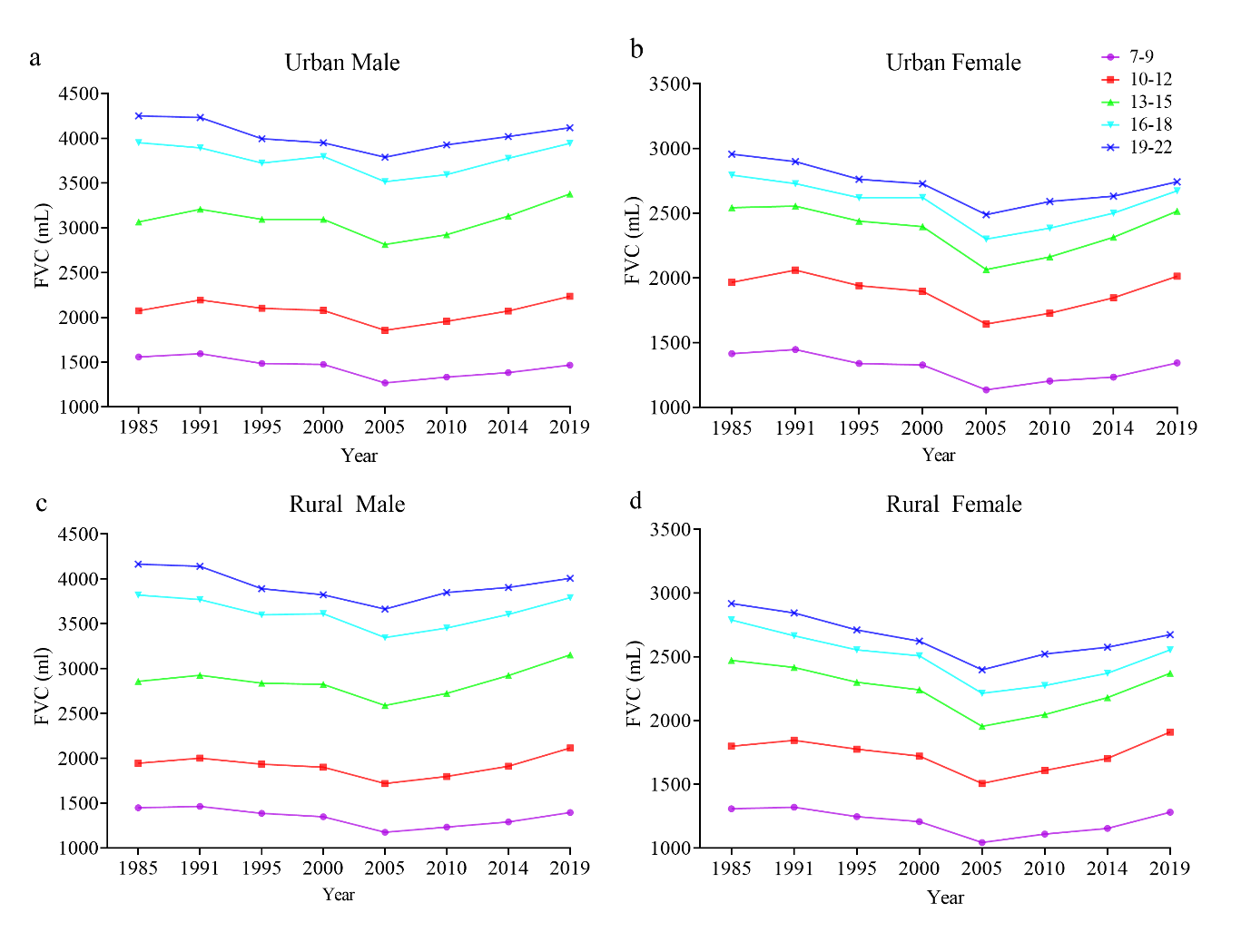


Figure S1 Trend in FVC in Chinese Han students from 1985 to 2019, by sex, region, and age groups.

Abbreviation: FVC, forced vital capacity.

Notes: a) Trend in FVC in urban male by age group, 1985 to 2019; b) Trend in FVC in urban female by age group, 1985 to 2019; c) Trend in FVC in rural male by age group, 1985 to 2019; d) Trend in FVC in rural female by age group, 1985 to 2019.


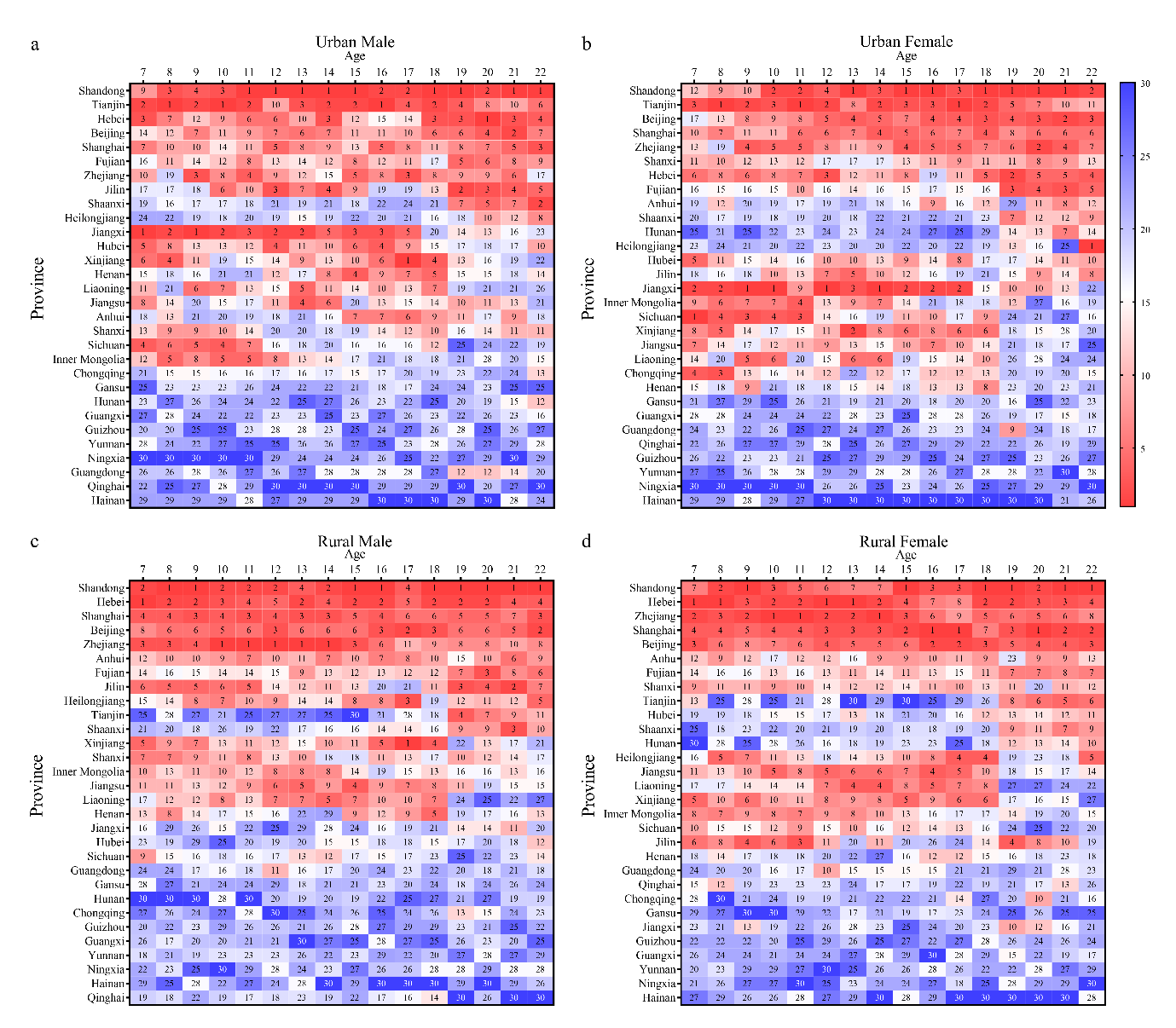


Figure S2 Ranking of FVC of Chinese Han students in 30 provinces in 2019, by sex and region.

Abbreviation: FVC, forced vital capacity.

Notes: a) Ranking of FVC in urban male of Han nationality in 30 Chinese provinces in 2019; b) Ranking of FVC in urban female of Han nationality in 30 Chinese provinces in 2019; c) Ranking of FVC in rural male of Han nationality in 30 Chinese provinces in 2019; d) Ranking of FVC in rural female of Han nationality in 30 Chinese provinces in 2019.


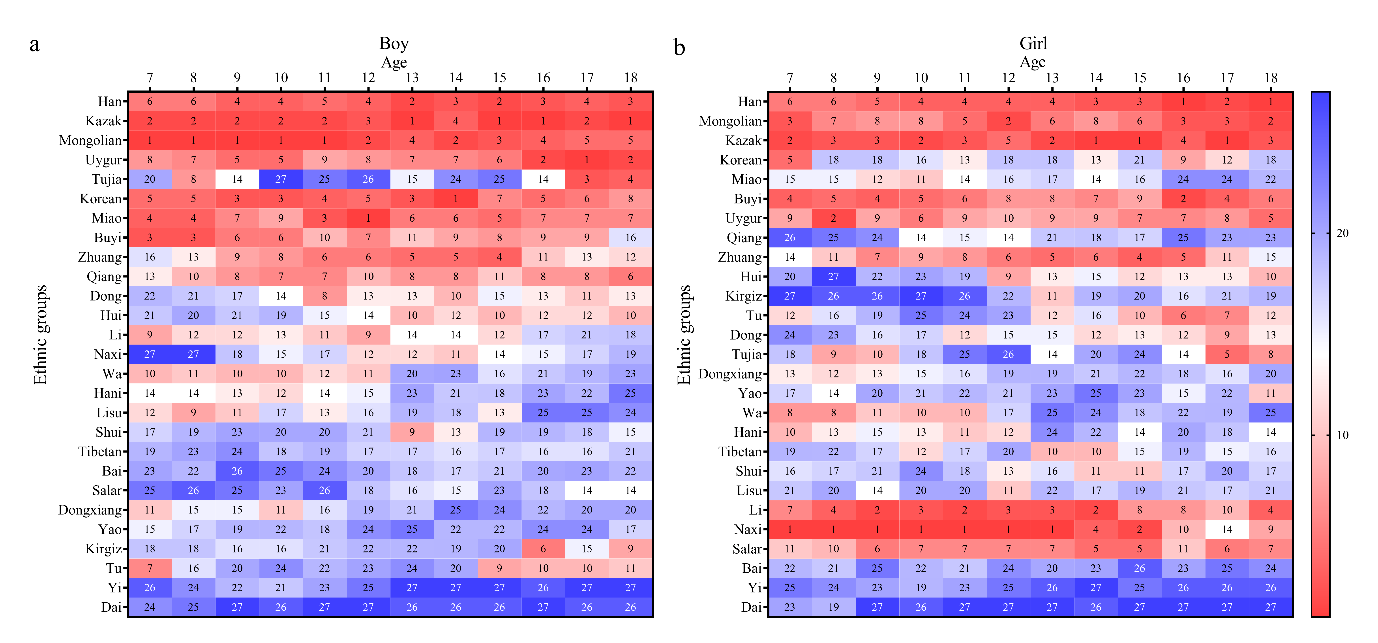


Figure S3 Ranking of FVC of Chinese students of 27 nationalities in 2019, by sex.

Abbreviation: FVC, forced vital capacity.

Notes: a) Ranking of FVC in boy from 27 Chinese nationalities in 2019; b) Ranking of FVC in girl from 27 Chinese nationalities in 2019.


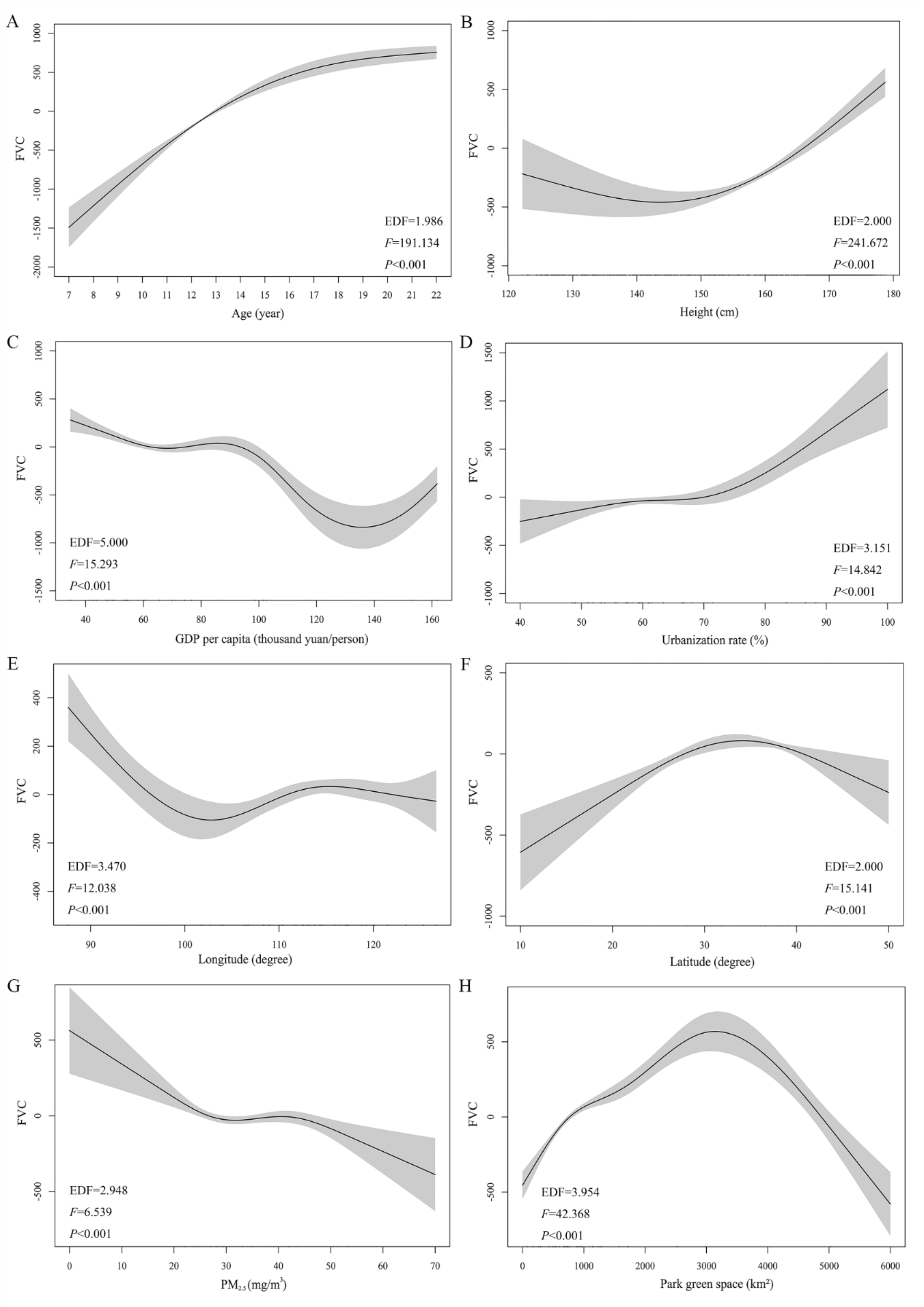


Figure S4: Relationships of influencing factors with Chinese male students' FVC in 2019 using GAM.

Abbreviations: FVC, forced vital capacity; GAM, generalized additive model; PM_2.5_, particulate matter with aerodynamics diameter <2.5 μm.

A) Effect of age on FVC in the multivariable GAM；B) Effect of height on FVC in the multivariable GAM**;** C) Effect of GDP per capita on FVC in the multivariable GAM**;** D) Effect of urbanization rate on FVC in the multivariable GAM; E) Effect of longitude on FVC in the multivariable GAM; F) Effect of latitude on FVC in the multivariable GAM; G) Effect of PM_2.5_ on FVC in the multivariable GAM; H) Effect of park green space on FVC in the multivariable GAM.

Notes: The values of PM_2.5_ in the table represented the annual mean PM_2.5_ value in 2019; the model was adjusted for area; adjusted R-Squared for this model was 0.962 and deviance explained was 96.2%.


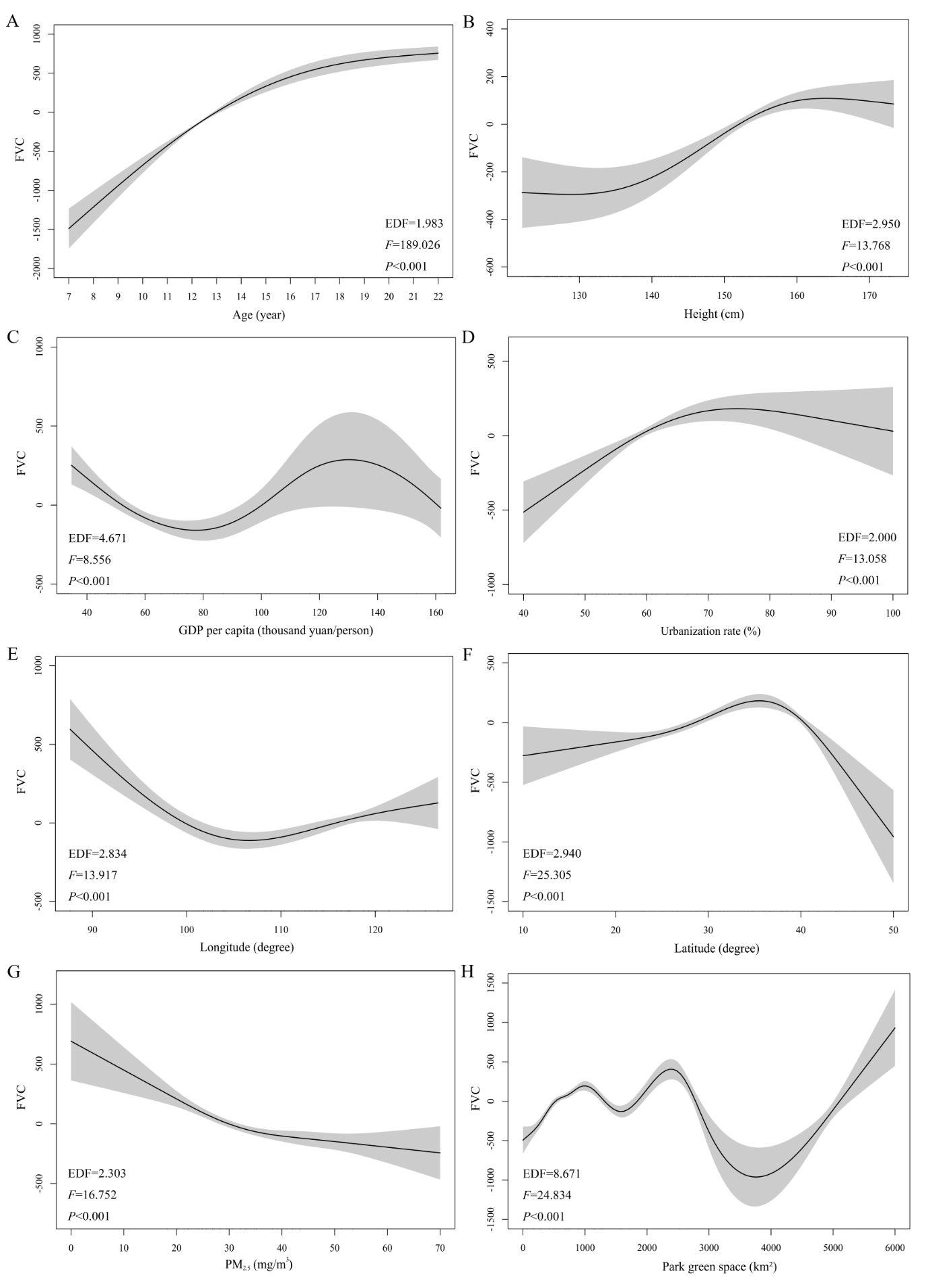
Figure S5: Relationships of influencing factors with Chinese female students' FVC in 2019 using GAM.

Abbreviations: FVC, forced vital capacity; GAM, generalized additive model; PM_2.5_, particulate matter with aerodynamics diameter <2.5 μm.

A) Effect of age on FVC in the multivariable GAM；B) Effect of height on FVC in the multivariable GAM**;** C) Effect of GDP per capita on FVC in the multivariable GAM**;** D) Effect of urbanization rate on FVC in the multivariable GAM; E) Effect of longitude on FVC in the multivariable GAM; F) Effect of latitude on FVC in the multivariable GAM; G) Effect of PM_2.5_ on FVC in the multivariable GAM; H) Effect of park green space on FVC in the multivariable GAM.

Notes: The values of PM_2.5_ in the table represented the annual mean PM_2.5_ value in 2019; the model was adjusted for area; adjusted R-Squared for this model was 0.907 and deviance explained was 90.7%.
